# Exosomal MiR-1290 Promotes Angiogenesis of Hepatocellular Carcinoma via Targeting SMEK1

**DOI:** 10.1155/2021/6617700

**Published:** 2021-01-29

**Authors:** Qiong Wang, Guanwen Wang, Lianjie Niu, Shaorong Zhao, Jianjun Li, Zhen Zhang, Huimin Jiang, Quansheng Zhang, Hang Wang, Peiqing Sun, Rong Xiang, Antao Chang, Shuang Yang

**Affiliations:** ^1^Tianjin Key Laboratory of Tumor Microenvironment and Neurovascular Regulation, Medical College of Nankai University, Tianjin 300071, China; ^2^Tianjin Medical University, Tianjin 300070, China; ^3^Tianjin Key Laboratory of Organ Transplantation, Tianjin First Central Hospital, Tianjin 300192, China; ^4^Department of Cancer Biology, Wake Forest University School of Medicine, Winston-Salem, NC 27157, USA

## Abstract

Hepatocellular carcinoma (HCC), the most common primary liver cancer, relies on the formation of new blood vessel for growth and frequent intrahepatic and extrahepatic metastasis. Therefore, it is important to explore the underlying molecular mechanisms of tumor angiogenesis of HCC. Recently, microRNAs have been shown to modulate angiogenic processes by modulating the expression of critical angiogenic factors. However, the potential roles of tumor-derived exosomal microRNAs in regulating tumor angiogenesis remain to be elucidated. In this study, our miRNome sequencing demonstrated that miR-1290 was overexpressed in HCC patient serum-derived exosomes, and we found that delivery of miR-1290 into human endothelial cells enhanced their angiogenic ability. Our results further revealed that SMEK1 is a direct target of miR-1290 in endothelial cells. MiR-1290 exerted its proangiogenic function, at least in part, by alleviating the inhibition of VEGFR2 phosphorylation done by SMEK1. Collectively, our findings provide evidence that miR-1290 is overexpressed in HCC and promotes tumor angiogenesis via exosomal secretion, implicating its potential role as a therapeutic target for HCC.

## 1. Introduction

Hepatocellular carcinoma is the fifth most common cancer and third-leading cause of cancer-related deaths worldwide [1]. HCC is a highly vascularized tumor with frequent intrahepatic and extrahepatic metastasis, which is responsible for the rapid recurrence and poor survival [2]. Therefore, it is urgent to explore the molecular mechanisms of tumor angiogenesis and metastasis, which would provide potential effective therapeutic approaches to improve the survival of HCC patients.

MicroRNAs (miRNAs) are a family of noncoding RNAs, about 22 nt in length, which repress gene expression by pairing to the 3′-untranslated regions of target messenger RNAs (mRNAs) [3]. MiRNAs are involved and play critical roles in a variety of biological processes such as tumor progression [4]. Deregulation of miRNAs contributes to angiogenesis and metastasis of various human cancers including HCC [5–7]. For example, microRNA-26a has been reported to suppress angiogenesis in HCC by targeting HGF-cMet pathway [8]. Recently, emerging evidence has revealed that miRNAs can be secreted into tumor microenvironment via exosomes to mediate the crosstalk between cancer cells and tumor microenvironment [9–12].

Exosomes are small (40–100 nm) membrane vesicles released into the extracellular environment [13]. It is reported that exosomes contain miRNAs selectively enriched from parent cells [14]. Circulating exosomal miRNAs may have additional advantages as biomarkers over and above ‘free' miRNAs. A number of plasma exosomal miRNAs are reported as diagnostic, prognostic, or even therapeutic biomarkers in cancer patients [15–17]. For example, exosomal miR-1290 and miR-375 are prognostic markers in castration-resistant prostate cancer [18]. Exosomal miR-210 could be delivered into endothelial cells and directly inhibits the expression of SMAD4 and STAT6, resulting in enhanced HCC angiogenesis [19]. However, the underlying mechanism of exosomal miRNAs in HCC development, especially in tumor angiogenesis of HCC, remains largely unknown.

In this study, by RNA sequencing and qRT-PCR verification, we revealed that miR-1290 was highly expressed in HCC patient serum-derived exosomes. Using gain- and loss-of-function analyses, we further demonstrated the proangiogenic function of miR-1290 in mediating the crosstalk between HCCs and tumor endothelial cells. Moreover, we found that SMEK1 was a *bona fide* target of miR-1290 in endothelial cells. MiR-1290 exerted its proangiogenic function, at least in part, by alleviating the inhibition of VEGFR2 phosphorylation done by SMEK1. Collectively, our findings suggest that exosomal miRNA-1290 might play an important role in the intercellular communication in tumor angiogenesis of HCC.

## 2. Materials and Methods

### 2.1. Clinical Specimens

Primary HCC tumor samples, paired tumor adjacent tissues, and serum samples of 49 HCC patients and serum samples of 28 healthy individuals were obtained from Tianjin First Central Hospital. Among them, serum samples from six HCC patients and two healthy individuals were subjected to miRNome sequencing. Serum samples and surgically removed tissues were frozen and transported in liquid nitrogen. All of the samples were obtained with written informed consent from patients and approval from the Ethics Committee of Tianjin First Central Hospital and Medical School of Nankai University.

### 2.2. Experimental Animals

Adult male BALB/*c* and NOD-SCID mice were ordered form HuaFuKang Bioscience Co. (Beijing, China), housed under specific pathogen free environmental conditions. All mouse experiments were conducted in accordance with the standard operating procedures approved by the Institute Research Ethics Committee at the Nankai University.

### 2.3. Exosome Purification

Exosome extraction from serum and cell supernatant samples using the exosome isolation reagent (Ribobio Co., China) were performed according to the manufacturer's protocol. For electron microscopy, the serum was precleared by centrifugation at 300 × *g* for 5 min, 2000 × *g* for 10 min, and then at 10000 × *g* for 60 min. Exosomes were isolated by ultracentrifugation at 100000 × *g* for 130 min, followed by PBS washing under the same ultracentrifugation conditions. Exosomes were resuspended in 100 *μ*l PBS, fixed with 2% paraformaldehyde, loaded on 200-mesh Formvar-coated grids, contrasted, and embedded for imaging.

### 2.4. Cell Lines and Reagents

Human umbilical vein endothelial cells (HUVECs) and human HCC cell lines Hep3 B and HepG2 were obtained from ATCC. HCC cell line SMMC-7721, PLC/PRF/5, and normal human hepatocyte cell line L-02 were purchased from the Chinese Academy of Sciences (Shanghai, China). Hep3 B, HepG2, and PLC/PRF/5 cells were cultured in MEM containing 10% fetal bovine serum (FBS), 100 U/ml penicillin/streptomycin, and 100 *μ*g/ml nonessential amino acids. SMMC-7721 cells were grown in DMEM containing 10% FBS and 100 U/ml penicillin/streptomycin. L-02 and HUVECs were cultured in RPMI-1640 containing 10% FBS and 100 U/ml penicillin/streptomycin. MiR-1290 mimics, inhibitors, agomir, antagomir, and miR-1290 mimic-FAM were designed and synthesized by Ribobio Co. The sequences of miR-1290 mimic and agomir are UGGAUUUUUGGAUCAGGGA and the sequences of miR-1290 inhibitor and antagomir are UCCCUGAUCCAAAAAUCCA. The miR-1290 agomir and antagomir were all nucleotides with a 2′-O-methyl modification. The miR-1290 mimic and inhibitor were transiently transfected with lipofectamine 2000 (Invitrogen) according to the manufacture's protocol.

### 2.5. Cell Viability Assays

For cell viability assays, HUVECs were seeded in 96-well plates in 100 *μ*l of medium, followed by transfection with 50 nM miR-1290 mimics. Five parallel wells were assigned for each group. At 0, 24, 36, 48, and 60 h after transfection, CCK-8 solution (10 *μ*l) was added to each well, and absorbance values were measured at 450 nm after incubation for 1.5 h at 37°C.

### 2.6. RNA Extraction and Quantitative Real-Time PCR (qRT-PCR)

Tissues were grinded using a homogenizer in Trizol reagent (Invitrogen, USA). Cells were collected, washed, and lysed in Trizol reagent, and total RNA was isolated following the manufacturer's instructions. qRT-PCR was performed using a standard SYBR-Green PCR kit protocol. Primers are listed in Table S1.

### 2.7. Western Blotting

Western blotting was performed in accordance with a previous protocol [20]. Briefly, proteins were loaded on 5–12% tris-acrylamide gels and membranes were blotted with specific antibodies. The primary antibodies used in this study were SMEK1 (ab70635, Abcam), pVEGFR2 (#4991, Cell Signaling Technology), and VEGFR2 (#9698, Cell Signaling Technology) at a dilution of 1 : 1000. Bands were detected using a Gel imaging system (SYNGENE).

### 2.8. Plasmid Construction and Transfection

Human SMEK1 cDNA was cloned from HUVECs and ligated into pLV-EF1*α*-MCS-IRES-Bsd vector (Biosettia Inc.). SMMC-7721, Hep3 B, and HUVECs (3 × 10^5^ cells/well) were seeded in 6-well plates, incubated overnight, and then transfected with miR-1290 mimics using lipofectamine 2000 (Invitrogen) and Opti-MEM (Corning) according to the manufacturer's instruction. Primers are listed in Table S2.

### 2.9. Cell Migration Assays

Cell migration was evaluated by performing wound-healing and transwell assays. For wound-healing assay, cells were seeded in 6-well plates at 2 × 10^5^ cells per well, and after 48 h transfection, the cell monolayer was scraped using a 10 *μ*l tip. The initial gap length and the residual gap length after wounding were calculated based on photomicrographs using ImageJ software. For transwell assay, after transfection for 24 h, cells were plated in 24-well plates at 5 × 10^4^ in the upper 8 *μ*m chambers (BD bioscience). Medium containing 10% FBS in the lower well and 2% FBS in the upper chamber served as the chemoattractant. After another 16 h incubation, cells on the upper surface of the membrane were scraped off and those on the bottom were stained with crystal violet. These cells were photographed using an optical microscope.

### 2.10. Tube Formation and Matrigel Plug Assays

For tube formation assay, a prechilled 48-well plate was coated with 150 *μ*l of Matrigel (BD bioscience) and incubated at 37°C for 30 min. HUVECs (3 × 10^4^ cells/well) were transfected with miR-1290 and then seeded on this plate. After 5 h, photographs were taken, and the tubes were counted.

For *in vivo* Matrigel plug assay, male BALB/*c* mice were administered a subcutaneous injection of 750 *μ*l of mixture containing 500 *μ*l Matrigel and 250 *μ*l EBM2 medium with or without 10 nmol miR-1290 agomir (3 mice for each group). After 10 days, the Matrigel plugs were imaged and snap frozen in the presence of optimum cutting temperature (OCT) medium before sectioning. Frozen Matrigel sections (8 *μ*m) were fixed in cold methanol and immunostained with a CD31 antibody (ab28364, Abcam, USA).

### 2.11. Immunofluorescence Staining

Slides were blocked with 2% BSA and then incubated with an anti-CD31 antibody (ab28364, Abcam) for 1 h. After fluorophore-conjugated secondary antibody incubating and PBS washing, slides were incubated with DAPI (Invitrogen) for 2 min. Images were obtained using a microscope (Olympus IX73).

### 2.12. Dual Luciferase Reporter Assay

HUVECs were cultured in 24-well plates at a density of 2 × 10^5^ cells/well overnight, which was followed by cotransfection with miR-1290 mimics, pmirGLO, and the pRL-TK plasmid. After 36 h of transfection, the inhibition of miR-1290 was quantified as the ratio of firefly luciferase activity to *Renilla* luciferase activity in each well.

### 2.13. Tumor Xenografts

Male NOD-SCID mice (6 weeks old) were separated randomly into two groups (*n* = 6 each), and 2 × 10^6^ SMMC-7721 cells were inoculated subcutaneously into each mouse, and tumors were allowed to grow for 10 days. Tumors were peritumorally treated with 10 nmol miR-1290 antagomir or miRNA antagomir negative control (NC) every three days, and tumor volume was measured with calipers once every 3 days.

### 2.14. Immunohistochemistry (IHC)

IHC staining was performed using paraffin-embedded human HCC tissues and mouse xenografts tumors. These tissues were probed separately with an antibody against SMEK1 (ab70635, Abcam), pVEGFR2 (#4991, Cell Signaling Technology), Ki67 (ab16667, Abcam), cleaved-caspase 3 (#9664, Cell Signaling Technology), and CD31 (ab28364, Abcam) at a 1 : 100 dilution.

### 2.15. Statistical Analysis

Statistical analyses were performed using SPSS 23.0 software; the data from all experiments are presented as means ± SD and represent three independent experiments. A paired *t*-test was used to compare gene expression in tumor and adjacent nontumor tissue samples. Where appropriate, a Student's *t*-test for unpaired observations was applied. A value of *p* < 0.05 was considered significant.

## 3. Results

### 3.1. MiR-1290 Is Highly Expressed in HCC Patient Serum-Derived Exosomes, Tumor Tissues, and HCC Cell Lines

**Figure 1:**
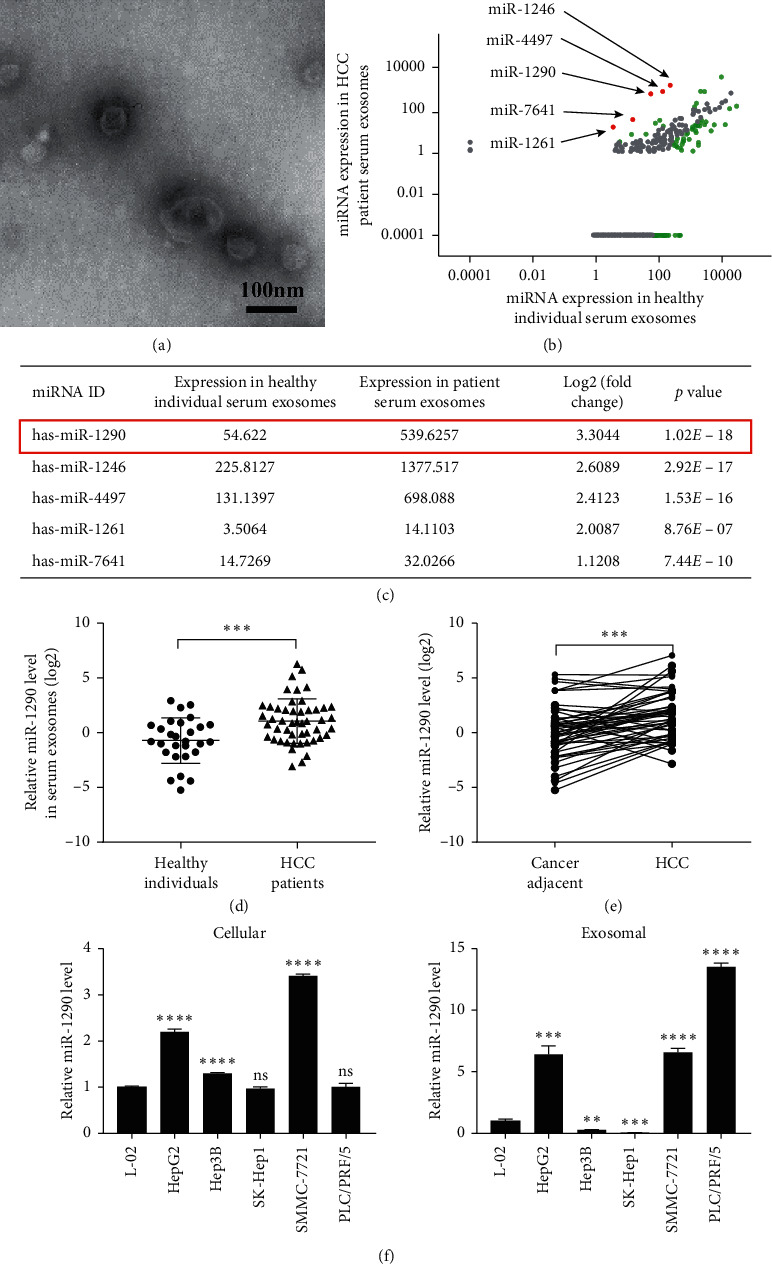
MiR-1290 is upregulated in serum-derived exosomes from HCC patients and cell lines. (a) Images of exosomes derived from HCC patient serum were captured by electron microscope. (b) The scatter diagram shows the differentially expressed miRNAs in exosomes derived from HCC patients and healthy individuals by RNA-sequencing. Red dots represent the upregulated miRNAs, green dots represent the downregulated miRNAs, and gray dots represent the unchanged miRNAs. (c) Detailed information of the significantly upregulated miRNAs are listed. (d, e) The expression levels of exosomal miR-1290 in serum from healthy individuals and HCC patients (d) and in HCC tissues and paired tumor adjacent tissues (e) were detected by qRT-PCR. (f) Cellular (left panel) and exosomal (right panel) miR-1290 levels in HCC and normal hepatic cell lines were assessed by qRT-PCR. ^*∗∗*^*p* < 0.01; ^*∗∗∗*^*p* < 0.001; ^*∗∗∗∗*^*p* < 0.0001. Ns, not significant.

To uncover miRNAs that are dysregulated in HCC patient serum-derived exosomes, we collected serum exosomes from the healthy individuals and HCC patients to perform RNA-seq (Figure 1(a)). We totally identified 599 differently expressed miRNAs (Table S3). After filtering (|log2 fold change|≥1, *p* value < 0.05), we acquired 5 upregulated and 72 downregulated miRNAs (Figure 1(b)). Since upregulated miRNAs are convenient biomarkers and potential therapeutic targets for HCC, we moved on to validate the upregulated miRNAs, including miR-1290, miR-1246, miR-4497, miR-1261, and miR-7641. Among these upregulated miRNAs, we pay our attention to miR-1290 because miR-1290 is the topmost upregulated miRNA (Figure 1(c)). To confirm the upregulation of miR-1290 in HCC patients, we further measured its expression in serum-derived exosomes from 28 healthy individuals and 49 HCC patients. The results revealed that the expression of miR-1290 was significantly increased in serum exosomes derived from 49 HCC patients as compared to those from 28 healthy individuals (Figure 1(d)). We also detected the expression level of miR-1290 in these 49 frozen HCC tissues and paired cancer adjacent tissues. Our qRT-PCR results demonstrated that miR-1290 expression was remarkably elevated in HCC tissues (Figure 1(e)). In addition, we assessed the expression of miR-1290 in 4 HCC cell lines, including HepG2, Hep3 B, SMMC-7721, and PLC/PRF/5 and their exosomes derived from the medium. An immortalized liver cell line L-02 was used as a control. Our results showed that the expression levels of miR-1290 were relatively higher in HepG2 and SMMC-7721 cells as well as their exosomes (Figure 1(f)). Taken together, the above observations indicated that miR-1290 is overexpressed in HCCs and cancer-cell-secreted exosomes.

### 3.2. HCC-Derived Exosomal MiR-1290 Targets Endothelial Cells to Promote Tumor Angiogenesis

**Figure 2:**
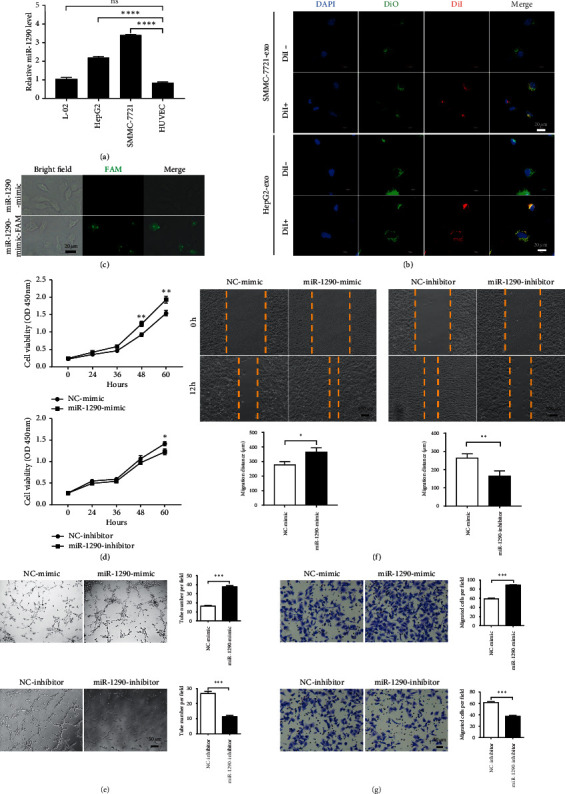
Exosomal miR-1290 targets endothelial cells and exerts an angiogenic effect *in vitro*. (a) The expression level of miR-1290 in HUVECs, HepG2, SMMC-7721, and L-02 cells were detected by qRT-PCR. (b) Exosomes were isolated from the conditioned medium of DiI-labeled (DiI+) or control (DiI-) HCCs and applied to culture with DiO-labeled HUVECs for 24 (h) followed by confocal microscope detection. (c) Exosomes were isolated from the conditioned medium of SMMC-7721 cells that transfected with miR-1290-FAM or miR-1290 mimic and applied to culture HUVECs for 6 h before detection for fluorescent FAM. (d) Cell viabilities of SMMC-7721 cellstransfected with 50 nM miR-1290 mimic, miR-1290 inhibitor, or the negative control were detected by CCK-8 assay. (e, f, g) The tube formation (e), wound-healing (f), and Transwell (g) assays of HUVECs were performed in HUVECs that transfected with 50 nM miR-1290 mimic, miR-1290 inhibitor, or the negative control. ^*∗*^*p* < 0.05; ^*∗∗*^*p* < 0.01; ^*∗∗∗*^*p* < 0.001; ^*∗∗∗∗*^*p* < 0.0001. Ns = not significant.

Next, we moved to validate whether HCC-derived exosomal miR-1290 could remodel the tumor microenvironment through targeting the endothelial cells and tumor angiogenesis. To this end, we firstly detected the expression of miR-1290 in HepG2, SMMC-7721, and HUVECs. As shown in Figure 2(a), the expression of miR-1290 was significantly increased in HepG2 and SMMC-7721 cells as compared to that in HUVECs (Figure 2(a)). To further test whether the cancer cell-derived exosomes can interact with HUVECs, we collected exosomes from HepG2 and SMMC-7721 cells labeled by DiI and incubated them with HUVECs labeled by DiO. We observed a colocalization of DiI and DiO signals in HUVECs cocultured with HCC-derived exosomes, indicating that cancer-cell-secreted exosomes can interact with endothelial cells (Figure 2(b)). We also analyzed whether miR-1290 can be transported from cancer cells to HUVECs as an exosome cargo. To do so, we transfected SMMC-7721 cells with FAM-labeled miR-1290 mimic. Exosomes were then derived from the medium to incubate with HUVECs. The results confirmed that FAM-labeled miR-1290 mimic were detected in HUVECs (Figure 2(c)), demonstrating that HCC-secreted miR-1290 can be transported from cancer cells to HUVECs via exosomes.

Additionally, we examined whether miR-1290 altered the function of HUVECs. Firstly, we performed CCK-8 assay to detect the cell viability of HUVECs in the presence of miR-1290 mimic and inhibitor, respectively. As shown in Figure 2(d), overexpression of miR-1290 mimic significantly enhanced cell viability at 48–60 h after transfection; however, miR-1290 inhibitor performed the opposite effect to reduce cell viability. Moreover, we measured the tube formation ability of HUVECs in response to miR-1290 mimic or inhibitor transfection. We found that the capacity of HUVECs to form tube-like structures on the Matrigel was increased by miR-1290 mimic but decreased by miR-1290 inhibitor (Figure 2(e)). Wound-healing and Transwell assays further revealed that miR-1290 mimic significantly accelerated HUVECs migration, whereas miR-1290 inhibitor decreased this effect (Figures 2(f) and 2(g)). These results collectively indicated that miR-1290 might promote endothelial function and tumor angiogenesis *in vitro*.

### 3.3. MiR-1290 Promotes Tumor Angiogenesis *In Vivo*

**Figure 3:**
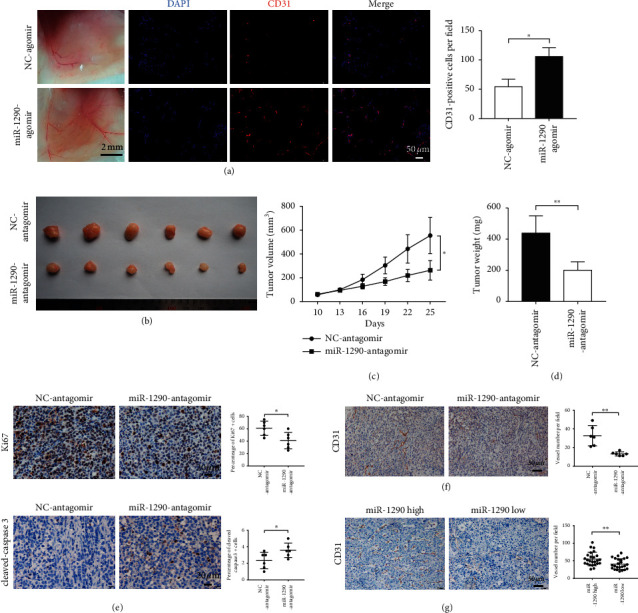
miR-1290 promotes tumor angiogenesis *in vivo*. (a) Matrigel mixed with miR-1290 agomir or NC agomir were subcutaneously injected into BALB/*c* mice (*n* = 3 each) and then harvested 10 days after inoculation, followed by immunofluorescence staining for CD31. (b) 2 × 10^6^ SMMC-7721 cells were subcutaneously injected into male NOD-SCID mice. Tumor development was allowed to occur for 10 days, and then mice (*n* = 6) were peritumorally treated with 10 nmol miR-1290 antagomir or NC antagomir (once every three days) for another 15 days. Tumors from miR-1290 antagomir and NC antagomir-treated mice are shown. (c, d) Tumor volume (c) and weight (d) were measured. The proliferation ((e), upper panel), apoptosis ((e), lower panel), and vessel density (f) of the tumors were assessed by IHC stanning for KI67, cleaved-caspase 3, and CD31 separately. (g) 49 HCC patients were divided into two groups based on their miR-1290 expression. The microvessel density of paraffin-embedded tumor sections from these 49 HCC patients was assessed by IHC stanning for CD31. ^*∗*^*p* < 0.05; ^*∗∗*^*p* < 0.01.

To determine whether miR-1290 is involved in tumor angiogenesis *in vivo*, Matrigel plug assays were performed in BALB/*c* mice. As shown in Figure 3(a), the numbers of newly generated blood vessels in Matrigel plugs were increased in mice treated with miR-1290 agomir, compared to the NC agomir group. Immunofluorescent staining of CD31 further revealed that treatment with miR-1290 agomir resulted in significantly increased microvessel density in the plug sections.

On the contrary, we established a SMMC-7721 xenograft tumor model in NOD-SCID nude mice using miR-1290 and NC antagomirs, respectively. The results indicated that tumor volumes and weights were significantly decreased in mice peritumorally injected with miR-1290 antagomir compared with those of mice injected with NC antagomir (Figures 3(a)–3(d)). Immunohistochemical analysis also revealed the downregulation of Ki67 and the upregulation of cleaved-caspase 3 in miR-1290 antagomir-treated tumors, indicating decreased cell proliferation and increased cell apoptosis (Figure 3(e)). Importantly, reduced angiogenesis was observed in miR-1290 antagomir-treated tumors, as assessed by CD31 staining (Figure 3(f)). Moreover, we divided 49 HCC patients into two groups based on their miR-1290 expression levels. Clinical association analysis showed that high level of miR-1290 was correlated with bigger tumor size and advanced clinical stages (Table 1). Notably, IHC staining for CD31 revealed that the microvessel density was increased in tumors with high miR-1290 expression compared to tumors with lower miR-1290 expression (Figure 3(g)), which is consistent with our results showing that miR-1290 acts as an oncogene to promote tumor angiogenesis *in vitro* and *in vivo*.

### 3.4. SMEK1 Is a Direct Target of MiR-1290 in HUVECs

**Figure 4:**
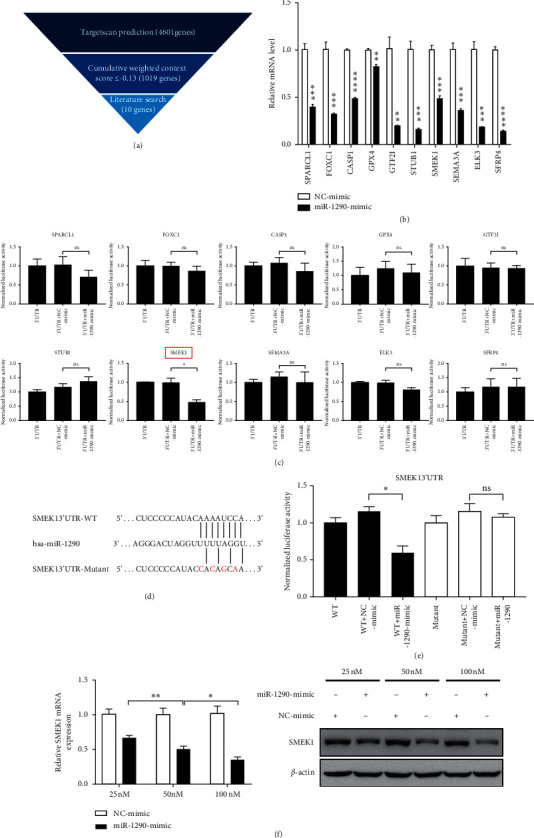
MiR-1290 targets SMEK1 directly in HUVECs. (a) Flow chart of screening miR-1290 target genes that were associated with angiogenesis. (b) The mRNA levels of 10 candidate genes in HUVECs that were transfected with miR-1290 mimic or NC mimic were detected by qRT-PCR. (c) HUVECs were cotransfected with miR-1290 mimic and the wild-type (WT) 3′UTR constructs of different candidate genes. Luciferase activities were determined 36 h after transfection. Luciferase values were normalized to Renilla activities. (d) The wild-type and mutant miR-1290 binding sites in the predicted target sequences of SMEK1 3′UTR are indicated. (e) HUVECs were cotransfected with miR-1290 mimic and WT or mutant SMEK1 3′UTR constructs. Luciferase activities were determined 36 h after transfection. Luciferase values were normalized to Renilla activities. (f) HUVECs were transfected with different concentrations of miR-1290 mimic for 48 h. The expression of SMEK1 was detected by qRT-PCR and Western blotting. ^*∗*^*p* < 0.05; ^*∗∗*^*p* < 0.01; ^*∗∗∗*^*p* < 0.001; ^*∗∗∗∗*^*p* < 0.0001. Ns = not significant.

To investigate the potential target genes of miR-1290 in the angiogenic process, we performed a miRNA target gene prediction with Targetscan database and identified 10 candidate genes that have been reported to function in the regulation of angiogenesis (Figure 4(a)). We then performed qRT-PCR to detect the mRNA levels of these 10 candidate genes in HUVECs transfected with miR-1290 mimic. The results demonstrated that all of these miR-1290 potential targets were downregulated by miR-1290 mimic transfection (Figure 4(b)). Moreover, we cloned the 3′UTR fragments of these genes into a pmirGLO luciferase reporter system, respectively. The results of luciferase assay showed a remarkable repression in the wild-type SMEK1 3′UTR reporter in response to miR-1290 mimic treatment in HUVECs (Figure 4(c)). However, this effect was significantly abolished by using the mutant SMEK1 3′UTR (Figures 4(d)–4(e)). To further confirm the correlation between miR-1290 and SMEK1, we detected the expression of SMEK1 in HUVECs upon transfection with miR-1290 mimic. Our results demonstrated that transfection of miR-1290 mimic downregulated SMEK1 expression at both the mRNA and protein levels in a dose-dependent manner (Figure 4(f)), suggesting that SMEK1 is a direct target of miR-1290. Western blot (Figure S1(a)) and immunohistochemical analysis also revealed the upregulation of SMEK1 (Figure S1(b)) and the downregulation of pVEGFR2 (Figure S1(c)) in the previous miR-1290 antagomir-treated xenografts.

### 3.5. SMEK1 Reduces the Angiogenic Ability of Endothelial Cells

**Figure 5:**
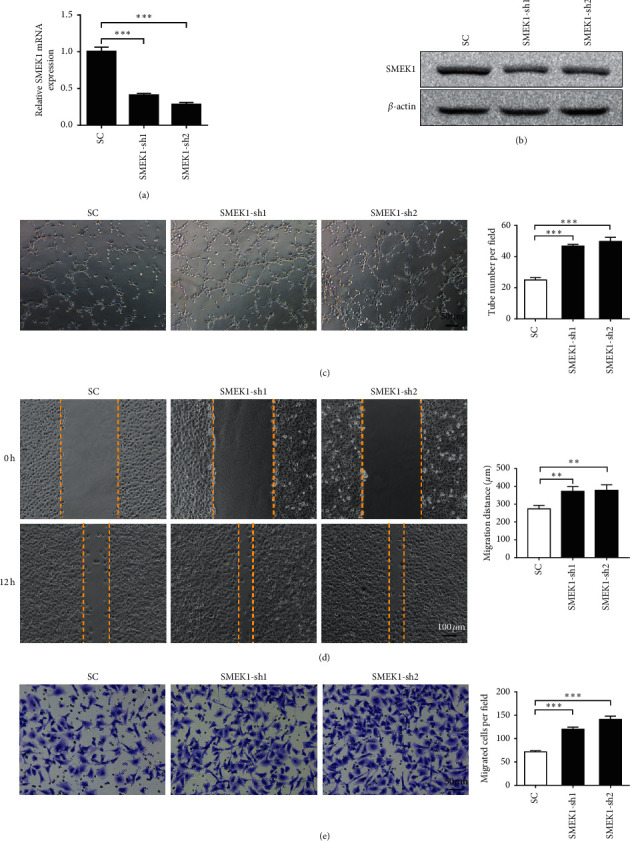
SMEK1 inhibits the angiogenic ability of endothelial cells. (a, b) Two independent shRNAs for SMEK1 were stably transfected into HUVECs via infection of lentivirus. The mRNA (a) and protein (b) level of SMEK1 were detected by qRT-PCR and Western blotting, respectively. (c, d, e) The tube formation assay (c), wound-healing assay (d), and Transwell assay (e) were performed in SMEK1-interfered HUVECs. ^*∗∗*^*p* < 0.01; ^*∗∗∗*^*p* < 0.001.

Considering that SMEK1 has been reported to function as an antiangiogenic factor by suppressing the phosphorylation of VEGFR2 in HUVECs [21], we further examined the endothelial phenotypes in response to SMEK1 interference. qRT-PCR and western blotting assays showed reduced SMEK1 mRNA and protein levels in HUVECs transfected with two independent SMEK1 shRNAs (Figures 5(a) and 5(b)). Notably, we found that the ability of HUVEC to form tube-like structures on the Matrigel was increased by SMEK1 knockdown (Figure 5(c)). Wound-healing and Transwell assays also revealed that depletion of SMEK1 significantly promoted the migration of HUVECs (Figures 5(d) and 5(e)), confirming that SMEK1 might act as a tumor suppressor to inhibit the endothelial function in HCC.

### 3.6. SMEK1 Mediates MiR-1290-Induced Proangiogenic Phenotypes

**Figure 6:**
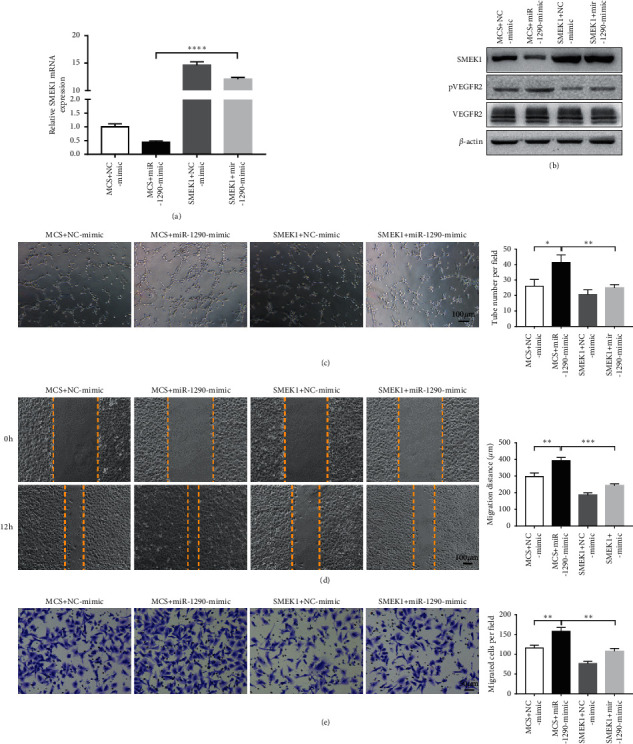
Rescue of SMEK1 expression attenuates the proangiogenic function of miR-1290. (a) SMEK1 were stably overexpressed in HUVECs in the presence or absence of miR-1290 mimic. The mRNA level of SMEK1 was detected by qRT-PCR. (b) The protein levels of SMEK1and pVEGFR2 were assessed by Western blotting. (c, d, e) The tube formation (c), wound-healing (d), and Transwell (e) assays were performed in SMEK1-expressing or control HUVECs in the presence or absence of miR-1290. ^*∗*^*p* < 0.05; ^*∗∗*^*p* < 0.01; ^*∗∗∗*^*p* < 0.001; ^*∗∗∗∗*^*p* < 0.0001.

Next, to study whether miR-1290 exerts its proangiogenic function through SMEK1, we overexpressed SMEK1 lacking 3′UTR in HUVECs via lentiviral transfection. As shown in Figures 6(a) and 6(b), overexpression of SMEK1 in HUVECs could restore the mRNA and protein levels of SMEK1 in the presence of miR-1290 mimic. Moreover, rescue of SMEK1 expression significantly attenuated the phosphorylation of VEGFR2 upon treatment with miR-1290 mimic (Figure 6(b)). Importantly, tube formation, wound-healing, and migration assays further demonstrated that restoration of SMEK1 abolished miR-1290 mimic-induced endothelial functions in HUVECs (Figures 6(c)–6(e)). These results collectively implied that SMEK1 is a *bona fide* target of miR-1290 to mediate its role in promoting tumor angiogenesis and progression.

## 4. Discussion

Tumor angiogenesis is responsible for growth, invasion, and metastasis of HCC [2]. A growing body of evidence has suggested that the dysfunction of miRNAs in HCC affects tumor angiogenesis [22–25]. In the present study, we demonstrate that miR-1290 exerts a proangiogenic function through SMEK1. We show that miR-1290 downregulates SMEK1 expression at both the mRNA and protein levels. Moreover, miR-1290 inhibits the luciferase activity of wild-type but not the mutant 3′UTR of SMEK1. By gain- and loss-of-function analyses, we further show that SMEK1 can reduce the angiogenic ability of endothelial cells. Moreover, restoration of SMEK1 expression significantly abolishes miR-1290-induced VEGFR2 phosphorylation and the angiogenic phenotypes of endothelial cells in HCC. According to our *in vivo* xenograft experiments, administration of the miR-1290 antagomir effectively reduced the tumor angiogenesis and progression, providing evidence that targeting miR-1290 might be a potential strategy for angiogenesis-based cancer therapy.

MiR-1290 has been reported to be a maker of several human cancers, including pancreatic cancer, esophageal squamous cell carcinoma, breast cancer, colorectal cancer, and non-small-cell lung cancer [26–30]. These studies have shown that miR-1290 functions as an oncogene to promote tumor progression. For example, a miRNA array analysis indicates that the detection of elevated serum miR-1290 has the potential to improve the early detection of pancreatic cancer [31]. Another study reveals that miR-1290 acts as a crucial driver for tumor initiation and progression in human non-small-cell lung cancer [32]. However, the function of miR-1290 in HCC remains largely obscure. In the present study, we extend the study that miR-1290 was highly expressed in HCC patient serum-derived exosomes, tumor tissues, and HCC cell lines. Moreover, the ectopic miR-1290 was secreted from tumor cells into its surrounding microenvironment and delivered into endothelial cells via exosomes. In turn, miR-1290 downregulated SMEK1 expression and enhanced the phosphorylation of VEGFR2 in endothelial cells, eventually exacerbating HCC development by promoting tumor angiogenesis. Our results are consistent with the previous reports that SMEK1 controls endothelial cell function and subsequent angiogenesis by suppressing VEGFR2-mediated PI3 K/Akt/eNOS signaling pathway. [21].

Given that VEGF functions as a critical factor to promote vascular abnormalities in HCC, numerous researchers have focused on the dysregulated miRNAs that can target VEGF or other genes that alter the VEGF expression. For example, miR-195 is reported to suppress angiogenesis of HCC by directly inhibiting the expression of VEGF [33]. Moreover, miR-29 c targets VEGFA to inhibit tumor angiogenesis of lung adenocarcinoma [34]. In this study, we described a novel pathway that tumor cells facilitated angiogenesis via secretion of exosomes containing miR-1290 into the surrounding microenvironment. Mechanistically, miR-1290 packed in exosomes were uptaken by recipient endothelial cells and subsequently downregulated SMEK1, thus resulting in enhanced tumor angiogenesis through a VEGFR2-mediated action. In line with this, tumor-derived exosomes have been shown to remodel the tumor and its metastatic environment [35–38]. Tumor cells can use exosomes as a cargo to transfer angiogenic factors, including proteins and miRNAs [39]. Recently, it has been demonstrated that exosomes released from human renal cancer stem cells trigger angiogenesis and form a prometastatic niche in lungs [40]. In line with these, our present study uncovered the function of secreted miR-1290 as a critical mediator in the crosstalk between HCCs and endothelial cells.

## 5. Conclusions

According to our data, mir-1290 is overexpressed in HCC patient serum-derived exosomes, and the delivery of miR-1290 into human endothelial cells enhanced their angiogenic ability by alleviating the inhibition of VEGFR2 phosphorylation done by SMEK1. Our findings highlight the importance of exosomal miRNA-1290 in HCC angiogenesis, implicating miR-1290 as a potential therapeutic target for HCC.

## Figures and Tables

**Table 1:** Correlation between the clinicopathological characteristics and miR-1290 expression in HCC.

Clinicopathologic features		*n*	Exosomal miR-1290		*p* value
			High	Low	
Gender	Male	37	18	19	0.202
	Female	12	7	5	
Age	<55	20	10	10	0.777
	≥55	29	15	14	
AFP	<400 *μ*g/L	23	10	13	0.157
	≥400 *μ*g/L	26	15	11	
TNM stage	I	16	6	10	0.004
	II + III	33	19	14	
Tumor size (cm)	<5	18	7	11	0.048
	≥5	31	18	13	

*Note.* The median expression level was used as the cut-off. Low expression of miR-1290 in 24 patients was classified as values below the 50th percentile. High miR-1290 expression in 25 patients was classified as values at or above the 50th percentile. For analysis of correlation between miR-1290 expressions and clinical features, chi-square tests were used. Results were considered statistically significant at *p* < 0.05

## Data Availability

The data used to support the findings of this study are available from the corresponding author upon request.

## Supplementary Materials

Supplementary Materials Figure S1. miR-1290 targets SMEK1 inSMMC-7721 xenografts Table S1. A list of primers used in the reactions for qRT-PCR. Table S2. A list of primers used in the reactions for clone PCR. Table S3. MiRNA sequencing results.
