# Supplementary material for: Exosomal MiR-1290 Promotes Angiogenesis of Hepatocellular Carcinoma via Targeting SMEK1
Source: J Oncol. 2021 Jan 29;2021:6617700. doi: 10.1155/2021/6617700 (PMC7864765; doi:10.1155/2021/6617700)
Supplement: Supplementary Materials — Figure S1. miR-1290 targets SMEK1 inSMMC-7721 xenografts Table S1. A list of primers used in the reactions for qRT-PCR. Table S2. A list of primers used in the reactions for clone PCR. Table S3. MiRNA sequencing results. [file 6617700.f1.zip › 6617700.f1/Fig. S1.docx]

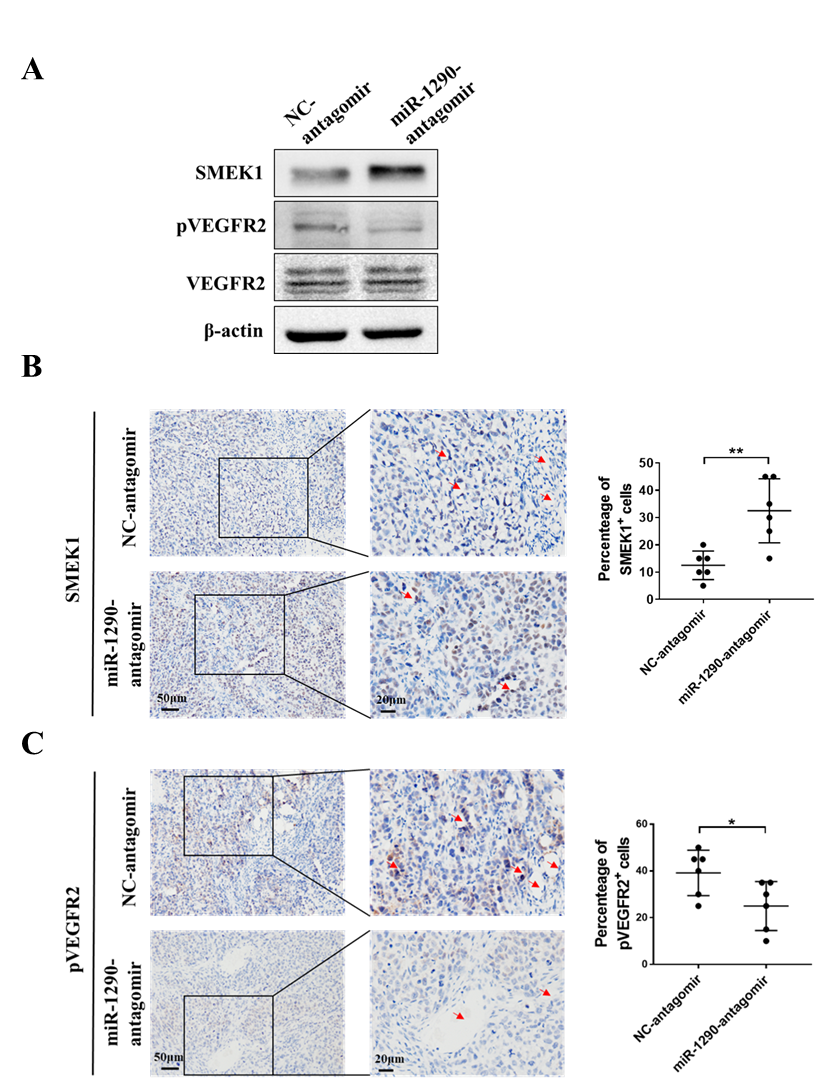


**Figure S1: miR-1290 targets SMEK1 in SMMC-7721 xenografts.** The tumor xenograft model was previously described in Figure 3**. A**, The expression of SMEK1 and pVEGFR2 in miR-1290 antagomir or NC antagomir treated xenografts were detected via Western blot. **B and C,** The expression of SMEK1 (**B**) and pVEGFR2 (**C**) in miR-1290 antagomir or NC antagomir treated xenografts were detected via IHC. Red arrow indicates blood vessel. **p* < 0.05; ***p* < 0.01.
