# Supplementary material for: Exosomal MiR-1290 Promotes Angiogenesis of Hepatocellular Carcinoma via Targeting SMEK1
Source: J Oncol. 2021 Jan 29;2021:6617700. doi: 10.1155/2021/6617700 (PMC7864765; doi:10.1155/2021/6617700)
Supplement: Supplementary Materials — Figure S1. miR-1290 targets SMEK1 inSMMC-7721 xenografts Table S1. A list of primers used in the reactions for qRT-PCR. Table S2. A list of primers used in the reactions for clone PCR. Table S3. MiRNA sequencing results. [file 6617700.f1.zip › 6617700.f1/Table S1.docx]

**Table S1. A list of primers used in the reactions for qRT-PCR.**

| **Real-time PCR primer (5’-3’):** | |
| --- | --- |
| U6-RT | AAAATATGGAACGCTTCACGAATTTG |
| U6-F | CTCGCTTCGGCACATATACT |
| U6-R | ACGCTTCACGAATTTGCGTGTC |
| miR-1290-RT | GTCGTATCCAGTGCAGGGTCCGAGGTATTCGCACTGGATCCCTG |
| miR-1290-F | CGCGCGTGGATTTTTGGAT |
| miR-1290-R | GTGCAGGGTCCGAGGT |
| GAPDH-F | GGAGCGAGATCCCTCCAAAAT |
| GAPDH-R | GGCTGTTGTCATACTTCTCATGG |
| SPARCL1-F | CCAACTGAAGGTACATTGGACAT |
| SPARCL1-R | CTGTGAAGGAACTAACACCAGG |
| FOXC1-F | CTGCCCGACTACTCTCTGC |
| FOXC1-R | CACCGAGTGGAAGTTCTGC |
| CASP1-F | TTTCCGCAAGGTTCGATTTTCA |
| CASP1-R | GGCATCTGCGCTCTACCATC |
| GPX4-F | GAGGCAAGACCGAAGTAAACTAC |
| GPX4-R | CCGAACTGGTTACACGGGAA |
| GTF2I-F | TTGTCGTCGGAACTGAAAGAG |
| GTF2I-R | CGATTTGCCTGGGTTGTAGAT |
| STUB1-F | AGCAGGGCAATCGTCTGTTC |
| STUB1-R | CAAGGCCCGGTTGGTGTAATA |
| SMEK1-F | ATTGTTGGCATGTTGCAGGAA |
| SMEK1-R | TTTTGAGGCTGTAGCGTTTG |
| SEMA3A-F | CTATCTTCCGAACTCTTGGGCA |
| SEMA3A-R | CTTTGGATCATTGAGCCACCT |
| ELK3-F | ATCTGCTGGACCTCGAACGA |
| ELK3-R | TTCTGCCCGATCACCTTCTTG |
| SFRP4-F | CACACCAGACATGATGGTACAG |
| SFRP4-R | GCTGAGATACGTTGCCAAAGTT |
