# Supplementary material for: Exosomal MiR-1290 Promotes Angiogenesis of Hepatocellular Carcinoma via Targeting SMEK1
Source: J Oncol. 2021 Jan 29;2021:6617700. doi: 10.1155/2021/6617700 (PMC7864765; doi:10.1155/2021/6617700)
Supplement: Supplementary Materials — Figure S1. miR-1290 targets SMEK1 inSMMC-7721 xenografts Table S1. A list of primers used in the reactions for qRT-PCR. Table S2. A list of primers used in the reactions for clone PCR. Table S3. MiRNA sequencing results. [file 6617700.f1.zip › 6617700.f1/Table S2.docx]

**Table S2. A list of primers used in the reactions for clone PCR.**

| **Used for subcloning and plasmid construction (5’-3’):** | |
| --- | --- |
| SMEK1-CDS-F | ATGACCGACACCCGGCGGCG |
| SMEK1-CDS-R | TTATTATGAATCAAATTTTGC |
| SPARCL1-3’UTR-clone-F | CCAACTGAAGGTACATTGGACAT |
| SPARCL1-3’UTR-clone-R | CTGTGAAGGAACTAACACCAGG |
| FOXC1-3’UTR-clone-F | CTGCCCGACTACTCTCTGC |
| FOXC1-3’UTR-clone-R | CACCGAGTGGAAGTTCTGC |
| CASP1-3’UTR-clone-F | TTTCCGCAAGGTTCGATTTTCA |
| CASP1-3’UTR-clone-R | GGCATCTGCGCTCTACCATC |
| GPX4-3’UTR-clone-F | GAGGCAAGACCGAAGTAAACTAC |
| GPX4-3’UTR-clone-R | CCGAACTGGTTACACGGGAA |
| GTF2I-3’UTR-clone-F | TTGTCGTCGGAACTGAAAGAG |
| GTF2I-3’UTR-clone-R | CGATTTGCCTGGGTTGTAGAT |
| STUB1-3’UTR-clone-F | AGCAGGGCAATCGTCTGTTC |
| STUB1-3’UTR-clone-R | CAAGGCCCGGTTGGTGTAATA |
| SMEK1-3’UTR-clone-F | ATTGTTGGCATGTTGCAGGAA |
| SMEK1-3’UTR-clone-R | TTTTGAGGCTGTAGCGTTTG |
| SEMA3A-3’UTR-clone-F | CTATCTTCCGAACTCTTGGGCA |
| SEMA3A-3’UTR-clone-R | CTTTGGATCATTGAGCCACCT |
| ELK3-3’UTR-clone-F | ATCTGCTGGACCTCGAACGA |
| ELK3-3’UTR-clone-R | TTCTGCCCGATCACCTTCTTG |
| SFRP4-3’UTR-clone-F | CACACCAGACATGATGGTACAG |
| SFRP4-3’UTR-clone-R | GCTGAGATACGTTGCCAAAGTT |
| SMEK1-CDS-clone-F | ATGACCGACACCCGGCGGCG |
| SMEK1-CDS-clone-F | TTATGAATCAAATTTTGCTT |
| SMEK1-shRNA-1 | AAAAGCAGAACTTGTGGCATCATCTTTGGATCCAAAGATGATGCCACAAGTTCTGC |
| SMEK1-shRNA-2 | AAAAGGAGGCACAACAGAATGATGATTGGATCCAATCATCATTCTGTTGTGCCTCC |
