# Supplementary material for: Exosomal MiR-1290 Promotes Angiogenesis of Hepatocellular Carcinoma via Targeting SMEK1
Source: J Oncol. 2021 Jan 29;2021:6617700. doi: 10.1155/2021/6617700 (PMC7864765; doi:10.1155/2021/6617700)
Supplement: Supplementary Materials — Figure S1. miR-1290 targets SMEK1 inSMMC-7721 xenografts Table S1. A list of primers used in the reactions for qRT-PCR. Table S2. A list of primers used in the reactions for clone PCR. Table S3. MiRNA sequencing results. [file 6617700.f1.zip › 6617700.f1/Table S3 miRNA sequencing results.docx]

| miRNA_ID | expression in healthy individual serum exosomes | expression in patient serum exosomes | log2  (fold change) | P-value | Significance-Lable |
| --- | --- | --- | --- | --- | --- |
| hsa-miR-122-5p | 9506.1053 | 3413.019 | 1.4778 | 4.83E-06 | ** |
| hsa-miR-1246 | 225.8127 | 1377.517 | 2.6089 | 2.92E-17 | ** |
| hsa-miR-378a-3p | 2094.728 | 717.0255 | 1.5466 | 7.68E-06 | ** |
| hsa-miR-4497 | 131.1397 | 698.088 | 2.4123 | 1.53E-16 | ** |
| hsa-miR-148a-3p | 2780.8151 | 683.6992 | 2.0241 | 0.000103879 | ** |
| hsa-miR-1290 | 54.622 | 539.6257 | 3.3044 | 1.02E-18 | ** |
| hsa-miR-22-3p | 17335.7581 | 534.7057 | 5.0189 | 0.666534848 |  |
| hsa-miR-320a | 11720.0512 | 228.3639 | 5.6815 | 0.155583907 |  |
| hsa-miR-100-5p | 1536.5858 | 192.7168 | 2.9952 | 0.010782195 | * |
| hsa-miR-146a-5p | 9019.4938 | 183.8051 | 5.6168 | 0.186672614 |  |
| hsa-miR-486-5p | 3060.3148 | 163.6608 | 4.2249 | 0.444393309 |  |
| hsa-miR-451a | 29018.6414 | 140.2674 | 7.6927 | 3.09E-05 | ** |
| hsa-miR-140-3p | 1341.7854 | 128.4779 | 3.3845 | 0.046655892 | * |
| hsa-let-7i-5p | 7532.7769 | 112.6967 | 6.0627 | 0.050350212 |  |
| hsa-miR-21-5p | 17346.4332 | 106.1057 | 7.353 | 0.00019872 | ** |
| hsa-miR-486-3p | 3368.0995 | 103.4136 | 5.0254 | 0.668419391 |  |
| hsa-miR-185-5p | 5513.3198 | 90.0459 | 5.9361 | 0.077110239 |  |
| hsa-miR-151a-3p | 2561.5478 | 87.7252 | 4.8679 | 0.850310582 |  |
| hsa-miR-143-3p | 1126.8815 | 85.8685 | 3.714 | 0.135092262 |  |
| hsa-miR-192-5p | 1306.4096 | 80.7628 | 4.0158 | 0.290496354 |  |
| hsa-miR-24-3p | 1654.6349 | 73.0579 | 4.5013 | 0.730373124 |  |
| hsa-miR-30d-5p | 3793.9332 | 69.6231 | 5.768 | 0.131354163 |  |
| hsa-miR-30a-5p | 1132.8814 | 58.4834 | 4.2758 | 0.506245378 |  |
| hsa-miR-103a-3p | 1097.7394 | 52.0781 | 4.3977 | 0.624610805 |  |
| hsa-miR-25-3p | 1933.4333 | 37.875 | 5.6737 | 0.180331668 |  |
| hsa-miR-134-5p | 423.0286 | 34.4402 | 3.6186 | 0.121629225 |  |
| hsa-miR-199a-3p | 478.8974 | 33.2334 | 3.849 | 0.219713424 |  |
| hsa-miR-7641 | 14.7269 | 32.0266 | 1.1208 | 7.44E-10 | ** |
| hsa-miR-152-3p | 105.8935 | 29.8915 | 1.8248 | 0.000171538 | ** |
| hsa-miR-101-3p | 2267.6329 | 29.2417 | 6.277 | 0.030935158 | * |
| hsa-miR-375 | 301.5511 | 28.2206 | 3.4176 | 0.074571149 |  |
| hsa-miR-191-5p | 3088.8336 | 24.8787 | 6.956 | 0.002331475 | ** |
| hsa-miR-3135b | 1534.0145 | 23.8575 | 6.0067 | 0.078689099 |  |
| hsa-let-7b-5p | 2052.5732 | 23.0221 | 6.4783 | 0.016818576 | * |
| hsa-miR-7704 | 76.5176 | 21.4439 | 1.8352 | 0.000405207 | ** |
| hsa-miR-26a-5p | 12657.6647 | 18.5662 | 9.4131 | 3.53E-09 | ** |
| hsa-miR-92a-3p | 2812.6066 | 18.2877 | 7.2649 | 0.00070152 | ** |
| hsa-miR-423-3p | 1507.3658 | 18.2877 | 6.365 | 0.02844028 | * |
| hsa-let-7a-5p | 5675.16 | 18.102 | 8.2923 | 3.47E-06 | ** |
| hsa-miR-629-5p | 239.4487 | 16.988 | 3.8172 | 0.236102328 |  |
| hsa-miR-186-5p | 1684.0108 | 16.7096 | 6.6551 | 0.010744316 | * |
| hsa-miR-27a-3p | 1373.0314 | 16.6167 | 6.3685 | 0.029118835 | * |
| hsa-miR-93-5p | 410.3276 | 16.5239 | 4.6341 | 0.898827861 |  |
| hsa-miR-30e-5p | 1682.1407 | 16.3382 | 6.6859 | 0.009556747 | ** |
| hsa-miR-1307-3p | 495.9619 | 16.1526 | 4.9404 | 0.816770021 |  |
| hsa-miR-3184-3p | 1956.5756 | 15.9669 | 6.9371 | 0.003390956 | ** |
| hsa-miR-320c | 135.1915 | 15.5956 | 3.1158 | 0.047692486 | * |
| hsa-miR-148b-3p | 642.296 | 15.0386 | 5.4165 | 0.387611741 |  |
| hsa-miR-181a-5p | 1165.53 | 14.296 | 6.3493 | 0.03484779 | * |
| hsa-miR-1261 | 3.5064 | 14.1103 | 2.0087 | 8.76E-07 | ** |
| hsa-miR-532-5p | 276.5387 | 13.4605 | 4.3606 | 0.634991192 |  |
| hsa-miR-10a-5p | 1208.6198 | 12.068 | 6.646 | 0.013604213 | * |
| hsa-miR-423-5p | 1134.9073 | 11.1397 | 6.6708 | 0.013013452 | * |
| hsa-miR-194-5p | 120.3087 | 11.0469 | 3.445 | 0.13135418 |  |
| hsa-miR-27b-3p | 236.5656 | 10.5827 | 4.4825 | 0.777582205 |  |
| hsa-let-7g-5p | 1453.0554 | 9.5616 | 7.2477 | 0.001664975 | ** |
| hsa-miR-744-5p | 718.7357 | 9.3759 | 6.2603 | 0.060597571 |  |
| hsa-miR-99a-5p | 198.2289 | 9.2831 | 4.4164 | 0.707955009 |  |
| hsa-miR-126-3p | 4506.747 | 9.1903 | 8.9378 | 2.24E-07 | ** |
| hsa-miR-28-3p | 317.9922 | 9.1903 | 5.1127 | 0.688905625 |  |
| hsa-miR-151a-5p | 91.3224 | 9.1903 | 3.3128 | 0.130877338 |  |
| hsa-miR-3184-5p | 599.6737 | 9.0974 | 6.0425 | 0.109691838 |  |
| hsa-miR-23a-3p | 418.1976 | 8.2619 | 5.6615 | 0.271312496 |  |
| hsa-miR-584-5p | 170.9569 | 7.8906 | 4.4374 | 0.747484665 |  |
| hsa-miR-221-3p | 1491.0025 | 7.7978 | 7.579 | 0.000506236 | ** |
| hsa-miR-106b-3p | 209.4494 | 7.705 | 4.7647 | 1 |  |
| hsa-miR-19a-3p | 327.5764 | 7.4265 | 5.463 | 0.418361926 |  |
| hsa-miR-146b-5p | 811.3048 | 7.3336 | 6.7896 | 0.012784528 | * |
| hsa-miR-7-5p | 213.6571 | 6.6838 | 4.9985 | 0.800481119 |  |
| hsa-miR-127-3p | 740.865 | 6.4053 | 6.8538 | 0.013411564 | * |
| hsa-miR-378b | 38.6484 | 6.4053 | 2.593 | 0.040093587 | * |
| hsa-miR-483-5p | 13.4023 | 6.3125 | 1.0862 | 0.002294842 | ** |
| hsa-miR-29a-3p | 488.2478 | 6.2197 | 6.2947 | 0.075025572 |  |
| hsa-miR-576-3p | 86.3356 | 6.034 | 3.8388 | 0.39180647 |  |
| hsa-miR-125b-5p | 139.3213 | 5.477 | 4.6689 | 1 |  |
| hsa-miR-99b-5p | 349.1603 | 5.477 | 5.9943 | 0.171798078 |  |
| hsa-miR-500a-3p | 69.1931 | 5.2914 | 3.709 | 0.37125267 |  |
| hsa-miR-125a-5p | 222.54 | 5.0129 | 5.4723 | 0.449741287 |  |
| hsa-miR-128-3p | 588.0635 | 4.7344 | 6.9566 | 0.013755349 | * |
| hsa-miR-17-5p | 130.5942 | 4.1774 | 4.9663 | 0.938413094 |  |
| hsa-miR-652-3p | 231.1892 | 4.1774 | 5.7903 | 0.316442819 |  |
| hsa-miR-382-5p | 100.9066 | 4.0846 | 4.6267 | 0.928217525 |  |
| hsa-miR-16-5p | 383.3673 | 3.9917 | 6.5856 | 0.047694224 | * |
| hsa-miR-370-3p | 597.9594 | 3.8989 | 7.2608 | 0.006491612 | ** |
| hsa-miR-199a-5p | 894.6794 | 3.7132 | 7.9125 | 0.000495617 | ** |
| hsa-miR-629-3p | 78.8552 | 3.6204 | 4.4449 | 0.832809174 |  |
| hsa-miR-223-3p | 209.9949 | 3.2491 | 6.0142 | 0.230584113 |  |
| hsa-miR-769-5p | 141.8147 | 3.2491 | 5.4479 | 0.526982887 |  |
| hsa-miR-363-3p | 599.6737 | 3.1562 | 7.5698 | 0.002970605 | ** |
| hsa-miR-335-5p | 23.6877 | 3.0634 | 2.951 | 0.238790282 |  |
| hsa-miR-340-5p | 579.3365 | 2.8778 | 7.6534 | 0.002960739 | ** |
| hsa-miR-223-5p | 62.0245 | 2.8778 | 4.4299 | 0.89852752 |  |
| hsa-miR-361-3p | 235.7085 | 2.8778 | 6.356 | 0.120781244 |  |
| hsa-miR-345-5p | 100.8287 | 2.8778 | 5.1309 | 0.767876888 |  |
| hsa-miR-409-3p | 352.1213 | 2.7849 | 6.9823 | 0.029877847 | * |
| hsa-miR-193b-5p | 5.6882 | 2.7849 | 1.0304 | 0.034782609 | * |
| hsa-miR-19b-3p | 68.1802 | 2.7849 | 4.6137 | 1 |  |
| hsa-miR-4448 | 17.532 | 2.6921 | 2.7032 | 0.251568225 |  |
| hsa-let-7f-5p | 1769.5671 | 2.5993 | 9.4111 | 7.27E-07 | ** |
| hsa-miR-181b-5p | 108.6207 | 2.5993 | 5.3851 | 0.700162868 |  |
| hsa-miR-660-5p | 41.3756 | 2.5993 | 3.9926 | 0.639220146 |  |
| hsa-miR-145-5p | 23.6098 | 2.5064 | 3.2357 | 0.390133159 |  |
| hsa-miR-5096 | 0 | 2.5064 | 14.6133 | 0.094202899 |  |
| hsa-miR-2110 | 73.6346 | 2.4136 | 4.9311 | 1 |  |
| hsa-miR-20a-5p | 283.0061 | 2.3208 | 6.9301 | 0.047590393 | * |
| hsa-miR-339-3p | 120.6204 | 2.3208 | 5.6997 | 0.458420067 |  |
| hsa-miR-378f | 4.9869 | 2.3208 | 1.1035 | 0.056521739 |  |
| hsa-miR-148a-5p | 55.9467 | 2.2279 | 4.6503 | 1 |  |
| hsa-miR-3615 | 142.9056 | 2.2279 | 6.0032 | 0.301975252 |  |
| hsa-miR-378g | 13.9477 | 2.2279 | 2.6463 | 0.266666667 |  |
| hsa-miR-425-5p | 539.4414 | 2.1351 | 7.981 | 0.001776582 | ** |
| hsa-miR-185-3p | 133.1656 | 2.0423 | 6.0269 | 0.315146324 |  |
| hsa-miR-144-3p | 1779.1513 | 1.9494 | 9.8339 | 1.92E-07 | ** |
| hsa-miR-142-5p | 301.0836 | 1.9494 | 7.2709 | 0.02167424 | * |
| hsa-miR-452-5p | 18.545 | 1.9494 | 3.2499 | 0.442578711 |  |
| hsa-miR-378d | 8.0258 | 1.9494 | 2.0415 | 0.186956522 |  |
| hsa-miR-941 | 26.2591 | 1.8566 | 3.822 | 0.670342248 |  |
| hsa-miR-1307-5p | 126.7761 | 1.8566 | 6.0934 | 0.293948602 |  |
| hsa-miR-3182 | 4.2077 | 1.8566 | 1.1803 | 0.094202899 |  |
| hsa-miR-3529-3p | 202.2808 | 1.8566 | 6.7675 | 0.086417525 |  |
| hsa-miR-30c-5p | 354.4589 | 1.7638 | 7.6508 | 0.009541561 | ** |
| hsa-miR-484 | 378.2245 | 1.5781 | 7.9049 | 0.00484733 | ** |
| hsa-let-7c-5p | 29.9213 | 1.5781 | 4.2449 | 1 |  |
| hsa-miR-1299 | 5.7661 | 1.5781 | 1.8694 | 0.282608696 |  |
| hsa-miR-4500 | 97.8677 | 1.5781 | 5.9546 | 0.4211558 |  |
| hsa-miR-27a-5p | 27.35 | 1.4853 | 4.2028 | 0.82249198 |  |
| hsa-miR-145-3p | 19.3242 | 1.4853 | 3.7016 | 0.585507246 |  |
| hsa-miR-222-3p | 479.287 | 1.3925 | 8.4271 | 0.00111463 | ** |
| hsa-miR-210-3p | 88.2056 | 1.3925 | 5.9852 | 0.454081825 |  |
| hsa-miR-130a-3p | 23.2981 | 1.3925 | 4.0645 | 0.79990005 |  |
| hsa-miR-330-3p | 42.8561 | 1.3925 | 4.9438 | 1 |  |
| hsa-miR-589-5p | 23.6877 | 1.3925 | 4.0885 | 0.79990005 |  |
| hsa-miR-30a-3p | 64.9854 | 1.2996 | 5.6439 | 0.662736074 |  |
| hsa-miR-182-5p | 182.0994 | 1.2996 | 7.1305 | 0.062845176 |  |
| hsa-miR-200b-3p | 92.1016 | 1.2996 | 6.1471 | 0.394949373 |  |
| hsa-miR-328-3p | 173.6061 | 1.2996 | 7.0616 | 0.070754028 |  |
| hsa-miR-501-3p | 16.8308 | 1.2996 | 3.6949 | 0.769726248 |  |
| hsa-miR-574-3p | 12.5451 | 1.2996 | 3.271 | 0.52173913 |  |
| hsa-miR-542-3p | 6.3115 | 1.2996 | 2.2799 | 0.430641822 |  |
| hsa-miR-4492 | 4.0518 | 1.2068 | 1.7474 | 0.285714286 |  |
| hsa-miR-142-3p | 516.2211 | 1.114 | 8.8561 | 0.00034089 | ** |
| hsa-miR-15a-5p | 46.5183 | 1.114 | 5.384 | 0.854436837 |  |
| hsa-miR-215-5p | 41.5315 | 1.114 | 5.2204 | 1 |  |
| hsa-miR-106b-5p | 123.5035 | 1.114 | 6.7927 | 0.147385428 |  |
| hsa-miR-485-5p | 19.0125 | 1.114 | 4.0932 | 1 |  |
| hsa-miR-664a-5p | 13.9477 | 1.114 | 3.6463 | 0.52173913 |  |
| hsa-miR-3653-3p | 0 | 1.114 | 13.4434 | 0.285714286 |  |
| hsa-miR-26b-5p | 1192.5683 | 1.0211 | 10.1897 | 8.28E-07 | ** |
| hsa-miR-885-5p | 3.8181 | 1.0211 | 1.9027 | 0.285714286 |  |
| hsa-miR-1180-3p | 67.0893 | 1.0211 | 6.0378 | 0.545915011 |  |
| hsa-miR-320d | 19.48 | 1.0211 | 4.2537 | 1 |  |
| hsa-miR-4791 | 0 | 1.0211 | 13.3179 | 0.523809524 |  |
| hsa-miR-129-5p | 11.9218 | 0.9283 | 3.6828 | 0.726708075 |  |
| hsa-miR-214-5p | 4.909 | 0.9283 | 2.4027 | 0.658385093 |  |
| hsa-miR-218-5p | 6.3895 | 0.9283 | 2.783 | 0.658385093 |  |
| hsa-miR-141-3p | 24.5449 | 0.9283 | 4.7246 | 1 |  |
| hsa-miR-200a-3p | 45.9729 | 0.9283 | 5.63 | 0.854436837 |  |
| hsa-miR-324-3p | 63.8166 | 0.9283 | 6.1032 | 0.431875402 |  |
| hsa-miR-146b-3p | 23.2981 | 0.9283 | 4.6494 | 1 |  |
| hsa-let-7d-5p | 472.8196 | 0 | 22.1728 | 8.62E-06 | ** |
| hsa-let-7d-3p | 204.0729 | 0 | 20.9606 | 0.000622182 | ** |
| hsa-miR-98-5p | 147.1912 | 0 | 20.4892 | 0.003964764 | ** |
| hsa-miR-106a-5p | 125.7632 | 0 | 20.2623 | 0.004729112 | ** |
| hsa-miR-16-2-3p | 451.3136 | 0 | 22.1057 | 1.21E-05 | ** |
| hsa-miR-10b-5p | 137.9966 | 0 | 20.3962 | 0.005759502 | ** |
| hsa-miR-144-5p | 184.4371 | 0 | 20.8147 | 0.001176447 | ** |
| hsa-miR-126-5p | 420.8469 | 0 | 22.0049 | 2.04E-05 | ** |
| hsa-miR-150-5p | 325.5505 | 0 | 21.6344 | 6.24E-05 | ** |
| hsa-miR-381-3p | 121.5555 | 0 | 20.2132 | 0.005858968 | ** |
| hsa-miR-326 | 160.9052 | 0 | 20.6178 | 0.002762764 | ** |
| hsa-miR-339-5p | 144.5419 | 0 | 20.463 | 0.004771142 | ** |
| hsa-miR-4433a-3p | 154.5157 | 0 | 20.5593 | 0.003304729 | ** |
| hsa-let-7a-3p | 80.4915 | 0 | 19.6185 | 0.038199172 | * |
| hsa-miR-139-5p | 81.6603 | 0 | 19.6393 | 0.038199172 | * |
| hsa-miR-183-5p | 102.8546 | 0 | 19.9722 | 0.01143183 | * |
| hsa-miR-224-5p | 67.7126 | 0 | 19.3691 | 0.029588432 | * |
| hsa-miR-140-5p | 88.5173 | 0 | 19.7556 | 0.023239034 | * |
| hsa-miR-200c-3p | 82.2837 | 0 | 19.6502 | 0.029721761 | * |
| hsa-miR-342-5p | 69.5048 | 0 | 19.4067 | 0.029588432 | * |
| hsa-miR-425-3p | 103.0884 | 0 | 19.9754 | 0.01143183 | * |
| hsa-miR-654-3p | 81.2707 | 0 | 19.6324 | 0.038199172 | * |
| hsa-miR-1301-3p | 66.6217 | 0 | 19.3456 | 0.039696281 | * |
| hsa-miR-543 | 78.2319 | 0 | 19.5774 | 0.038199172 | * |
| hsa-let-7a-2-3p | 0.8571 | 0 | 13.0653 | 1 |  |
| hsa-let-7b-3p | 18.2333 | 0 | 17.4762 | 0.430641822 |  |
| hsa-let-7e-5p | 38.8042 | 0 | 18.5658 | 0.191275791 |  |
| hsa-let-7f-1-3p | 2.4934 | 0 | 14.6058 | 1 |  |
| hsa-let-7f-2-3p | 2.3376 | 0 | 14.5127 | 1 |  |
| hsa-miR-16-1-3p | 2.8051 | 0 | 14.7758 | 1 |  |
| hsa-miR-17-3p | 31.8694 | 0 | 18.2818 | 0.186956522 |  |
| hsa-miR-18a-5p | 15.1165 | 0 | 17.2058 | 0.658385093 |  |
| hsa-miR-18a-3p | 5.844 | 0 | 15.8347 | 1 |  |
| hsa-miR-21-3p | 38.6484 | 0 | 18.56 | 0.191275791 |  |
| hsa-miR-22-5p | 57.4272 | 0 | 19.1314 | 0.053632861 |  |
| hsa-miR-23a-5p | 5.9219 | 0 | 15.8538 | 1 |  |
| hsa-miR-24-1-5p | 0.7792 | 0 | 12.9278 | 1 |  |
| hsa-miR-24-2-5p | 45.8171 | 0 | 18.8055 | 0.13787392 |  |
| hsa-miR-25-5p | 12.3893 | 0 | 16.9187 | 1 |  |
| hsa-miR-26b-3p | 14.0256 | 0 | 17.0977 | 0.658385093 |  |
| hsa-miR-28-5p | 37.8692 | 0 | 18.5307 | 0.191275791 |  |
| hsa-miR-31-5p | 1.1688 | 0 | 13.5127 | 1 |  |
| hsa-miR-32-5p | 28.6746 | 0 | 18.1294 | 0.186956522 |  |
| hsa-miR-32-3p | 1.6363 | 0 | 13.9982 | 1 |  |
| hsa-miR-33a-5p | 11.6101 | 0 | 16.825 | 1 |  |
| hsa-miR-92a-1-5p | 6.7791 | 0 | 16.0488 | 1 |  |
| hsa-miR-93-3p | 10.5192 | 0 | 16.6827 | 1 |  |
| hsa-miR-95-5p | 1.013 | 0 | 13.3063 | 1 |  |
| hsa-miR-95-3p | 5.2986 | 0 | 15.6933 | 1 |  |
| hsa-miR-96-5p | 23.6877 | 0 | 17.8538 | 0.282608696 |  |
| hsa-miR-98-3p | 2.2597 | 0 | 14.4638 | 1 |  |
| hsa-miR-101-5p | 0.935 | 0 | 13.1908 | 1 |  |
| hsa-miR-29b-3p | 34.5966 | 0 | 18.4003 | 0.124959742 |  |
| hsa-miR-29b-2-5p | 4.5973 | 0 | 15.4885 | 1 |  |
| hsa-miR-103a-2-5p | 0.8571 | 0 | 13.0653 | 1 |  |
| hsa-miR-107 | 1.948 | 0 | 14.2497 | 1 |  |
| hsa-miR-196a-5p | 7.3245 | 0 | 16.1604 | 1 |  |
| hsa-miR-197-3p | 33.7394 | 0 | 18.3641 | 0.124959742 |  |
| hsa-miR-30c-2-3p | 2.6493 | 0 | 14.6933 | 1 |  |
| hsa-miR-30d-3p | 14.5711 | 0 | 17.1527 | 0.658385093 |  |
| hsa-miR-139-3p | 32.3369 | 0 | 18.3028 | 0.124959742 |  |
| hsa-miR-147a | 5.5323 | 0 | 15.7556 | 1 |  |
| hsa-miR-10a-3p | 9.1167 | 0 | 16.4762 | 0.523809524 |  |
| hsa-miR-10b-3p | 2.0259 | 0 | 14.3063 | 1 |  |
| hsa-miR-34a-5p | 1.3246 | 0 | 13.6933 | 1 |  |
| hsa-miR-181a-2-3p | 34.4407 | 0 | 18.3937 | 0.124959742 |  |
| hsa-miR-181c-5p | 6.7011 | 0 | 16.0321 | 1 |  |
| hsa-miR-181c-3p | 2.5714 | 0 | 14.6502 | 1 |  |
| hsa-miR-187-3p | 0.8571 | 0 | 13.0653 | 1 |  |
| hsa-miR-199b-5p | 16.597 | 0 | 17.3406 | 0.658385093 |  |
| hsa-miR-203a-3p | 13.4023 | 0 | 17.0321 | 0.658385093 |  |
| hsa-miR-204-5p | 2.4155 | 0 | 14.56 | 1 |  |
| hsa-miR-204-3p | 2.961 | 0 | 14.8538 | 1 |  |
| hsa-miR-205-5p | 9.8959 | 0 | 16.5945 | 0.523809524 |  |
| hsa-miR-181a-3p | 43.8691 | 0 | 18.7428 | 0.13787392 |  |
| hsa-miR-219a-5p | 0.7792 | 0 | 12.9278 | 1 |  |
| hsa-miR-219a-1-3p | 1.4026 | 0 | 13.7758 | 1 |  |
| hsa-miR-221-5p | 13.636 | 0 | 17.0571 | 0.658385093 |  |
| hsa-miR-200b-5p | 2.5714 | 0 | 14.6502 | 1 |  |
| hsa-let-7g-3p | 1.7922 | 0 | 14.1294 | 1 |  |
| hsa-let-7i-3p | 17.2983 | 0 | 17.4003 | 0.658385093 |  |
| hsa-miR-1-3p | 8.9608 | 0 | 16.4513 | 0.523809524 |  |
| hsa-miR-15b-5p | 10.5971 | 0 | 16.6933 | 1 |  |
| hsa-miR-15b-3p | 43.8691 | 0 | 18.7428 | 0.13787392 |  |
| hsa-miR-23b-5p | 1.5584 | 0 | 13.9278 | 1 |  |
| hsa-miR-23b-3p | 46.3625 | 0 | 18.8226 | 0.13787392 |  |
| hsa-miR-27b-5p | 7.792 | 0 | 16.2497 | 1 |  |
| hsa-miR-30b-5p | 45.1158 | 0 | 18.7833 | 0.13787392 |  |
| hsa-miR-30b-3p | 3.1947 | 0 | 14.9634 | 1 |  |
| hsa-miR-125b-1-3p | 1.1688 | 0 | 13.5127 | 1 |  |
| hsa-miR-132-5p | 1.8701 | 0 | 14.1908 | 1 |  |
| hsa-miR-132-3p | 2.3376 | 0 | 14.5127 | 1 |  |
| hsa-miR-133a-3p | 7.0907 | 0 | 16.1136 | 1 |  |
| hsa-miR-135a-5p | 1.1688 | 0 | 13.5127 | 1 |  |
| hsa-miR-138-5p | 1.1688 | 0 | 13.5127 | 1 |  |
| hsa-miR-143-5p | 15.7399 | 0 | 17.2641 | 0.658385093 |  |
| hsa-miR-191-3p | 11.6101 | 0 | 16.825 | 1 |  |
| hsa-miR-125a-3p | 3.5064 | 0 | 15.0977 | 1 |  |
| hsa-miR-127-5p | 6.7791 | 0 | 16.0488 | 1 |  |
| hsa-miR-136-5p | 15.0386 | 0 | 17.1983 | 0.658385093 |  |
| hsa-miR-136-3p | 32.259 | 0 | 18.2993 | 0.186956522 |  |
| hsa-miR-146a-3p | 10.2075 | 0 | 16.6393 | 0.523809524 |  |
| hsa-miR-150-3p | 22.6748 | 0 | 17.7907 | 0.282608696 |  |
| hsa-miR-154-5p | 1.8701 | 0 | 14.1908 | 1 |  |
| hsa-miR-186-3p | 2.6493 | 0 | 14.6933 | 1 |  |
| hsa-miR-188-5p | 2.0259 | 0 | 14.3063 | 1 |  |
| hsa-miR-190a-5p | 4.5194 | 0 | 15.4638 | 1 |  |
| hsa-miR-195-5p | 3.3506 | 0 | 15.0321 | 1 |  |
| hsa-miR-195-3p | 2.0259 | 0 | 14.3063 | 1 |  |
| hsa-miR-206 | 3.2726 | 0 | 14.9982 | 1 |  |
| hsa-miR-155-5p | 47.2975 | 0 | 18.8514 | 0.099975819 |  |
| hsa-miR-194-3p | 1.0909 | 0 | 13.4132 | 1 |  |
| hsa-miR-29c-5p | 8.805 | 0 | 16.426 | 0.523809524 |  |
| hsa-miR-29c-3p | 8.805 | 0 | 16.426 | 0.523809524 |  |
| hsa-miR-30c-1-3p | 4.2856 | 0 | 15.3872 | 1 |  |
| hsa-miR-200a-5p | 2.0259 | 0 | 14.3063 | 1 |  |
| hsa-miR-219a-2-3p | 1.0909 | 0 | 13.4132 | 1 |  |
| hsa-miR-34b-5p | 1.2467 | 0 | 13.6058 | 1 |  |
| hsa-miR-34c-5p | 2.8051 | 0 | 14.7758 | 1 |  |
| hsa-miR-301a-5p | 2.883 | 0 | 14.8153 | 1 |  |
| hsa-miR-301a-3p | 1.6363 | 0 | 13.9982 | 1 |  |
| hsa-miR-99b-3p | 16.5191 | 0 | 17.3338 | 0.658385093 |  |
| hsa-miR-296-3p | 3.1947 | 0 | 14.9634 | 1 |  |
| hsa-miR-130b-5p | 29.298 | 0 | 18.1604 | 0.186956522 |  |
| hsa-miR-130b-3p | 9.8179 | 0 | 16.5831 | 0.523809524 |  |
| hsa-miR-30e-3p | 35.2199 | 0 | 18.426 | 0.124959742 |  |
| hsa-miR-26a-2-3p | 1.013 | 0 | 13.3063 | 1 |  |
| hsa-miR-361-5p | 30.3109 | 0 | 18.2095 | 0.186956522 |  |
| hsa-miR-362-5p | 8.6491 | 0 | 16.4003 | 0.523809524 |  |
| hsa-miR-365a-3p | 1.4026 | 0 | 13.7758 | 1 |  |
| hsa-miR-376c-3p | 8.7271 | 0 | 16.4132 | 0.523809524 |  |
| hsa-miR-369-5p | 4.6752 | 0 | 15.5127 | 1 |  |
| hsa-miR-369-3p | 11.3763 | 0 | 16.7957 | 1 |  |
| hsa-miR-374a-5p | 40.9081 | 0 | 18.642 | 0.191275791 |  |
| hsa-miR-374a-3p | 10.0517 | 0 | 16.6171 | 0.523809524 |  |
| hsa-miR-376a-3p | 4.5973 | 0 | 15.4885 | 1 |  |
| hsa-miR-377-5p | 5.3765 | 0 | 15.7144 | 1 |  |
| hsa-miR-377-3p | 9.1167 | 0 | 16.4762 | 0.523809524 |  |
| hsa-miR-378a-5p | 7.4024 | 0 | 16.1757 | 1 |  |
| hsa-miR-379-5p | 41.0639 | 0 | 18.6475 | 0.191275791 |  |
| hsa-miR-379-3p | 4.5973 | 0 | 15.4885 | 1 |  |
| hsa-miR-382-3p | 3.9739 | 0 | 15.2783 | 1 |  |
| hsa-miR-383-5p | 0.935 | 0 | 13.1908 | 1 |  |
| hsa-miR-340-3p | 28.4409 | 0 | 18.1176 | 0.186956522 |  |
| hsa-miR-330-5p | 10.2855 | 0 | 16.6502 | 0.523809524 |  |
| hsa-miR-342-3p | 44.0249 | 0 | 18.7479 | 0.13787392 |  |
| hsa-miR-337-5p | 1.1688 | 0 | 13.5127 | 1 |  |
| hsa-miR-337-3p | 3.7402 | 0 | 15.1908 | 1 |  |
| hsa-miR-323a-5p | 1.0909 | 0 | 13.4132 | 1 |  |
| hsa-miR-323a-3p | 13.3243 | 0 | 17.0237 | 0.658385093 |  |
| hsa-miR-148b-5p | 11.9997 | 0 | 16.8726 | 1 |  |
| hsa-miR-331-5p | 1.7922 | 0 | 14.1294 | 1 |  |
| hsa-miR-331-3p | 9.1946 | 0 | 16.4885 | 0.523809524 |  |
| hsa-miR-324-5p | 14.649 | 0 | 17.1604 | 0.658385093 |  |
| hsa-miR-338-5p | 21.0384 | 0 | 17.6827 | 0.430641822 |  |
| hsa-miR-338-3p | 2.0259 | 0 | 14.3063 | 1 |  |
| hsa-miR-335-3p | 52.1286 | 0 | 18.9917 | 0.072973421 |  |
| hsa-miR-196b-5p | 3.1168 | 0 | 14.9278 | 1 |  |
| hsa-miR-422a | 3.5843 | 0 | 15.1294 | 1 |  |
| hsa-miR-424-5p | 6.4674 | 0 | 15.9809 | 1 |  |
| hsa-miR-424-3p | 26.6487 | 0 | 18.0237 | 0.282608696 |  |
| hsa-miR-20b-5p | 21.5839 | 0 | 17.7196 | 0.430641822 |  |
| hsa-miR-429 | 12.7789 | 0 | 16.9634 | 0.658385093 |  |
| hsa-miR-450a-5p | 20.1034 | 0 | 17.6171 | 0.430641822 |  |
| hsa-miR-431-5p | 9.2725 | 0 | 16.5007 | 0.523809524 |  |
| hsa-miR-431-3p | 1.4026 | 0 | 13.7758 | 1 |  |
| hsa-miR-433-3p | 45.5054 | 0 | 18.7957 | 0.13787392 |  |
| hsa-miR-329-5p | 1.0909 | 0 | 13.4132 | 1 |  |
| hsa-miR-329-3p | 10.5192 | 0 | 16.6827 | 1 |  |
| hsa-miR-409-5p | 9.5842 | 0 | 16.5484 | 0.523809524 |  |
| hsa-miR-412-5p | 1.8701 | 0 | 14.1908 | 1 |  |
| hsa-miR-410-3p | 14.1036 | 0 | 17.1057 | 0.658385093 |  |
| hsa-miR-483-3p | 2.883 | 0 | 14.8153 | 1 |  |
| hsa-miR-485-3p | 17.532 | 0 | 17.4196 | 0.658385093 |  |
| hsa-miR-487a-5p | 1.2467 | 0 | 13.6058 | 1 |  |
| hsa-miR-487a-3p | 1.0909 | 0 | 13.4132 | 1 |  |
| hsa-miR-490-3p | 2.961 | 0 | 14.8538 | 1 |  |
| hsa-miR-491-5p | 1.2467 | 0 | 13.6058 | 1 |  |
| hsa-miR-511-5p | 6.5453 | 0 | 15.9982 | 1 |  |
| hsa-miR-493-5p | 39.7393 | 0 | 18.6002 | 0.191275791 |  |
| hsa-miR-493-3p | 46.1287 | 0 | 18.8153 | 0.13787392 |  |
| hsa-miR-432-5p | 39.8951 | 0 | 18.6058 | 0.191275791 |  |
| hsa-miR-494-3p | 19.1684 | 0 | 17.5484 | 0.430641822 |  |
| hsa-miR-495-3p | 28.7525 | 0 | 18.1333 | 0.186956522 |  |
| hsa-miR-497-5p | 4.4414 | 0 | 15.4387 | 1 |  |
| hsa-miR-181d-5p | 35.6874 | 0 | 18.445 | 0.124959742 |  |
| hsa-miR-499a-5p | 9.9738 | 0 | 16.6058 | 0.523809524 |  |
| hsa-miR-501-5p | 0.7792 | 0 | 12.9278 | 1 |  |
| hsa-miR-502-3p | 14.4932 | 0 | 17.145 | 0.658385093 |  |
| hsa-miR-503-5p | 9.8179 | 0 | 16.5831 | 0.523809524 |  |
| hsa-miR-505-5p | 10.5971 | 0 | 16.6933 | 1 |  |
| hsa-miR-505-3p | 18.0775 | 0 | 17.4638 | 0.430641822 |  |
| hsa-miR-532-3p | 3.3506 | 0 | 15.0321 | 1 |  |
| hsa-miR-455-5p | 5.1427 | 0 | 15.6502 | 1 |  |
| hsa-miR-539-3p | 6.5453 | 0 | 15.9982 | 1 |  |
| hsa-miR-487b-3p | 7.9479 | 0 | 16.2783 | 0.523809524 |  |
| hsa-miR-552-3p | 1.4026 | 0 | 13.7758 | 1 |  |
| hsa-miR-92b-5p | 2.1818 | 0 | 14.4132 | 1 |  |
| hsa-miR-92b-3p | 21.1164 | 0 | 17.688 | 0.430641822 |  |
| hsa-miR-556-5p | 1.8701 | 0 | 14.1908 | 1 |  |
| hsa-miR-574-5p | 1.8701 | 0 | 14.1908 | 1 |  |
| hsa-miR-576-5p | 4.4414 | 0 | 15.4387 | 1 |  |
| hsa-miR-579-5p | 2.1038 | 0 | 14.3607 | 1 |  |
| hsa-miR-580-3p | 1.948 | 0 | 14.2497 | 1 |  |
| hsa-miR-582-3p | 2.2597 | 0 | 14.4638 | 1 |  |
| hsa-miR-548a-3p | 2.8051 | 0 | 14.7758 | 1 |  |
| hsa-miR-550a-3p | 1.948 | 0 | 14.2497 | 1 |  |
| hsa-miR-590-3p | 4.5973 | 0 | 15.4885 | 1 |  |
| hsa-miR-598-3p | 8.9608 | 0 | 16.4513 | 0.523809524 |  |
| hsa-miR-548a-5p | 0.8571 | 0 | 13.0653 | 1 |  |
| hsa-miR-610 | 0.7792 | 0 | 12.9278 | 1 |  |
| hsa-miR-615-3p | 2.2597 | 0 | 14.4638 | 1 |  |
| hsa-miR-624-5p | 1.2467 | 0 | 13.6058 | 1 |  |
| hsa-miR-625-5p | 1.013 | 0 | 13.3063 | 1 |  |
| hsa-miR-625-3p | 36.3887 | 0 | 18.4731 | 0.124959742 |  |
| hsa-miR-627-5p | 1.948 | 0 | 14.2497 | 1 |  |
| hsa-miR-627-3p | 1.013 | 0 | 13.3063 | 1 |  |
| hsa-miR-628-5p | 1.7922 | 0 | 14.1294 | 1 |  |
| hsa-miR-628-3p | 8.6491 | 0 | 16.4003 | 0.523809524 |  |
| hsa-miR-33b-5p | 0.8571 | 0 | 13.0653 | 1 |  |
| hsa-miR-636 | 1.013 | 0 | 13.3063 | 1 |  |
| hsa-miR-641 | 3.6622 | 0 | 15.1604 | 1 |  |
| hsa-miR-642a-3p | 1.1688 | 0 | 13.5127 | 1 |  |
| hsa-miR-651-5p | 6.7791 | 0 | 16.0488 | 1 |  |
| hsa-miR-411-5p | 21.0384 | 0 | 17.6827 | 0.430641822 |  |
| hsa-miR-411-3p | 2.6493 | 0 | 14.6933 | 1 |  |
| hsa-miR-654-5p | 26.4149 | 0 | 18.011 | 0.282608696 |  |
| hsa-miR-656-3p | 2.8051 | 0 | 14.7758 | 1 |  |
| hsa-miR-659-5p | 1.013 | 0 | 13.3063 | 1 |  |
| hsa-miR-660-3p | 2.8051 | 0 | 14.7758 | 1 |  |
| hsa-miR-421 | 14.4152 | 0 | 17.1372 | 0.658385093 |  |
| hsa-miR-758-3p | 15.9736 | 0 | 17.2853 | 0.658385093 |  |
| hsa-miR-671-5p | 9.4283 | 0 | 16.5247 | 0.523809524 |  |
| hsa-miR-671-3p | 15.584 | 0 | 17.2497 | 0.658385093 |  |
| hsa-miR-550a-3-5p | 6.6232 | 0 | 16.0152 | 1 |  |
| hsa-miR-1224-5p | 3.6622 | 0 | 15.1604 | 1 |  |
| hsa-miR-151b | 5.4544 | 0 | 15.7351 | 1 |  |
| hsa-miR-320b | 14.7269 | 0 | 17.1681 | 0.658385093 |  |
| hsa-miR-1296-5p | 1.4026 | 0 | 13.7758 | 1 |  |
| hsa-miR-1468-5p | 3.2726 | 0 | 14.9982 | 1 |  |
| hsa-miR-1271-5p | 1.2467 | 0 | 13.6058 | 1 |  |
| hsa-miR-454-5p | 2.883 | 0 | 14.8153 | 1 |  |
| hsa-miR-454-3p | 5.2986 | 0 | 15.6933 | 1 |  |
| hsa-miR-766-5p | 13.2464 | 0 | 17.0152 | 0.658385093 |  |
| hsa-miR-766-3p | 12.3893 | 0 | 16.9187 | 1 |  |
| hsa-miR-1185-1-3p | 23.7657 | 0 | 17.8585 | 0.282608696 |  |
| hsa-miR-1298-5p | 1.0909 | 0 | 13.4132 | 1 |  |
| hsa-miR-300 | 33.8953 | 0 | 18.3707 | 0.124959742 |  |
| hsa-miR-450b-5p | 6.7791 | 0 | 16.0488 | 1 |  |
| hsa-miR-874-3p | 18.2333 | 0 | 17.4762 | 0.430641822 |  |
| hsa-miR-889-3p | 10.4413 | 0 | 16.6719 | 0.523809524 |  |
| hsa-miR-708-3p | 1.4026 | 0 | 13.7758 | 1 |  |
| hsa-miR-190b | 2.1818 | 0 | 14.4132 | 1 |  |
| hsa-miR-744-3p | 1.4805 | 0 | 13.8538 | 1 |  |
| hsa-miR-877-5p | 3.3506 | 0 | 15.0321 | 1 |  |
| hsa-miR-877-3p | 1.013 | 0 | 13.3063 | 1 |  |
| hsa-miR-665 | 1.0909 | 0 | 13.4132 | 1 |  |
| hsa-miR-873-5p | 2.4155 | 0 | 14.56 | 1 |  |
| hsa-miR-873-3p | 1.5584 | 0 | 13.9278 | 1 |  |
| hsa-miR-374b-5p | 25.2461 | 0 | 17.9457 | 0.282608696 |  |
| hsa-miR-374b-3p | 1.2467 | 0 | 13.6058 | 1 |  |
| hsa-miR-760 | 20.5709 | 0 | 17.6502 | 0.430641822 |  |
| hsa-miR-939-5p | 6.3895 | 0 | 15.9634 | 1 |  |
| hsa-miR-940 | 1.5584 | 0 | 13.9278 | 1 |  |
| hsa-miR-942-5p | 9.6621 | 0 | 16.56 | 0.523809524 |  |
| hsa-miR-942-3p | 2.4155 | 0 | 14.56 | 1 |  |
| hsa-miR-1228-5p | 2.4155 | 0 | 14.56 | 1 |  |
| hsa-miR-1229-3p | 1.0909 | 0 | 13.4132 | 1 |  |
| hsa-miR-548e-3p | 7.9479 | 0 | 16.2783 | 0.523809524 |  |
| hsa-miR-548j-3p | 0.7792 | 0 | 12.9278 | 1 |  |
| hsa-miR-1285-3p | 11.9218 | 0 | 16.8632 | 1 |  |
| hsa-miR-1287-5p | 21.2722 | 0 | 17.6986 | 0.430641822 |  |
| hsa-miR-1294 | 3.896 | 0 | 15.2497 | 1 |  |
| hsa-miR-1297 | 29.4538 | 0 | 18.1681 | 0.186956522 |  |
| hsa-miR-1303 | 1.013 | 0 | 13.3063 | 1 |  |
| hsa-miR-1304-5p | 1.2467 | 0 | 13.6058 | 1 |  |
| hsa-miR-1304-3p | 6.857 | 0 | 16.0653 | 1 |  |
| hsa-miR-548f-5p | 2.8051 | 0 | 14.7758 | 1 |  |
| hsa-miR-548f-3p | 1.7142 | 0 | 14.0653 | 1 |  |
| hsa-miR-1250-5p | 7.8699 | 0 | 16.2641 | 0.523809524 |  |
| hsa-miR-1254 | 2.0259 | 0 | 14.3063 | 1 |  |
| hsa-miR-1255a | 1.4805 | 0 | 13.8538 | 1 |  |
| hsa-miR-548o-3p | 2.8051 | 0 | 14.7758 | 1 |  |
| hsa-miR-1268a | 3.1168 | 0 | 14.9278 | 1 |  |
| hsa-miR-1277-3p | 3.1947 | 0 | 14.9634 | 1 |  |
| hsa-miR-1278 | 0.7792 | 0 | 12.9278 | 1 |  |
| hsa-miR-1284 | 1.6363 | 0 | 13.9982 | 1 |  |
| hsa-miR-1292-5p | 3.9739 | 0 | 15.2783 | 1 |  |
| hsa-miR-1255b-5p | 2.4934 | 0 | 14.6058 | 1 |  |
| hsa-miR-1255b-2-3p | 1.1688 | 0 | 13.5127 | 1 |  |
| hsa-miR-664a-3p | 1.6363 | 0 | 13.9982 | 1 |  |
| hsa-miR-1306-5p | 1.013 | 0 | 13.3063 | 1 |  |
| hsa-miR-1306-3p | 7.8699 | 0 | 16.2641 | 0.523809524 |  |
| hsa-miR-1197 | 1.4026 | 0 | 13.7758 | 1 |  |
| hsa-miR-1538 | 0.935 | 0 | 13.1908 | 1 |  |
| hsa-miR-103b | 5.2986 | 0 | 15.6933 | 1 |  |
| hsa-miR-1827 | 1.1688 | 0 | 13.5127 | 1 |  |
| hsa-miR-1908-5p | 12.7789 | 0 | 16.9634 | 0.658385093 |  |
| hsa-miR-1976 | 3.3506 | 0 | 15.0321 | 1 |  |
| hsa-miR-2277-5p | 1.013 | 0 | 13.3063 | 1 |  |
| hsa-miR-2277-3p | 0.8571 | 0 | 13.0653 | 1 |  |
| hsa-miR-2278 | 0.7792 | 0 | 12.9278 | 1 |  |
| hsa-miR-3120-5p | 1.948 | 0 | 14.2497 | 1 |  |
| hsa-miR-3120-3p | 4.909 | 0 | 15.5831 | 1 |  |
| hsa-miR-3124-5p | 1.1688 | 0 | 13.5127 | 1 |  |
| hsa-miR-3127-3p | 1.4026 | 0 | 13.7758 | 1 |  |
| hsa-miR-1273c | 1.8701 | 0 | 14.1908 | 1 |  |
| hsa-miR-3074-5p | 15.584 | 0 | 17.2497 | 0.658385093 |  |
| hsa-miR-3074-3p | 1.013 | 0 | 13.3063 | 1 |  |
| hsa-miR-3158-3p | 39.5834 | 0 | 18.5945 | 0.191275791 |  |
| hsa-miR-3161 | 0.935 | 0 | 13.1908 | 1 |  |
| hsa-miR-1260b | 4.2077 | 0 | 15.3607 | 1 |  |
| hsa-miR-3168 | 2.4155 | 0 | 14.56 | 1 |  |
| hsa-miR-3173-5p | 0.7792 | 0 | 12.9278 | 1 |  |
| hsa-miR-323b-3p | 12.9347 | 0 | 16.9809 | 0.658385093 |  |
| hsa-miR-3176 | 2.883 | 0 | 14.8153 | 1 |  |
| hsa-miR-3177-3p | 5.2986 | 0 | 15.6933 | 1 |  |
| hsa-miR-3065-5p | 2.1818 | 0 | 14.4132 | 1 |  |
| hsa-miR-3187-3p | 2.6493 | 0 | 14.6933 | 1 |  |
| hsa-miR-320e | 27.9733 | 0 | 18.0937 | 0.186956522 |  |
| hsa-miR-3196 | 0.8571 | 0 | 13.0653 | 1 |  |
| hsa-miR-3200-5p | 1.4026 | 0 | 13.7758 | 1 |  |
| hsa-miR-3200-3p | 1.2467 | 0 | 13.6058 | 1 |  |
| hsa-miR-4306 | 31.3239 | 0 | 18.2569 | 0.186956522 |  |
| hsa-miR-4317 | 0.7792 | 0 | 12.9278 | 1 |  |
| hsa-miR-4326 | 5.1427 | 0 | 15.6502 | 1 |  |
| hsa-miR-2355-5p | 1.5584 | 0 | 13.9278 | 1 |  |
| hsa-miR-2355-3p | 27.6617 | 0 | 18.0775 | 0.186956522 |  |
| hsa-miR-4286 | 10.2855 | 0 | 16.6502 | 0.523809524 |  |
| hsa-miR-3605-3p | 1.3246 | 0 | 13.6933 | 1 |  |
| hsa-miR-3611 | 3.1947 | 0 | 14.9634 | 1 |  |
| hsa-miR-3614-5p | 1.013 | 0 | 13.3063 | 1 |  |
| hsa-miR-3656 | 11.9997 | 0 | 16.8726 | 1 |  |
| hsa-miR-3677-3p | 1.3246 | 0 | 13.6933 | 1 |  |
| hsa-miR-3688-5p | 1.4805 | 0 | 13.8538 | 1 |  |
| hsa-miR-3688-3p | 0.7792 | 0 | 12.9278 | 1 |  |
| hsa-miR-3180 | 2.0259 | 0 | 14.3063 | 1 |  |
| hsa-miR-3909 | 0.935 | 0 | 13.1908 | 1 |  |
| hsa-miR-3912-3p | 1.4026 | 0 | 13.7758 | 1 |  |
| hsa-miR-3913-5p | 3.5064 | 0 | 15.0977 | 1 |  |
| hsa-miR-3913-3p | 4.0518 | 0 | 15.3063 | 1 |  |
| hsa-miR-3922-5p | 2.3376 | 0 | 14.5127 | 1 |  |
| hsa-miR-3928-3p | 7.7141 | 0 | 16.2352 | 1 |  |
| hsa-miR-3934-5p | 2.3376 | 0 | 14.5127 | 1 |  |
| hsa-miR-548y | 3.5843 | 0 | 15.1294 | 1 |  |
| hsa-miR-3940-3p | 0.935 | 0 | 13.1908 | 1 |  |
| hsa-miR-548ab | 2.1818 | 0 | 14.4132 | 1 |  |
| hsa-miR-4429 | 13.0127 | 0 | 16.9895 | 0.658385093 |  |
| hsa-miR-548ad-5p | 7.0907 | 0 | 16.1136 | 1 |  |
| hsa-miR-548ad-3p | 0.935 | 0 | 13.1908 | 1 |  |
| hsa-miR-4433a-5p | 59.1414 | 0 | 19.1738 | 0.053632861 |  |
| hsa-miR-548ae-3p | 1.0909 | 0 | 13.4132 | 1 |  |
| hsa-miR-4443 | 3.1947 | 0 | 14.9634 | 1 |  |
| hsa-miR-4446-3p | 23.2981 | 0 | 17.8298 | 0.282608696 |  |
| hsa-miR-378h | 1.013 | 0 | 13.3063 | 1 |  |
| hsa-miR-548aj-5p | 3.0389 | 0 | 14.8912 | 1 |  |
| hsa-miR-4466 | 0.935 | 0 | 13.1908 | 1 |  |
| hsa-miR-4470 | 1.948 | 0 | 14.2497 | 1 |  |
| hsa-miR-3155b | 1.013 | 0 | 13.3063 | 1 |  |
| hsa-miR-548ak | 0.8571 | 0 | 13.0653 | 1 |  |
| hsa-miR-4488 | 2.6493 | 0 | 14.6933 | 1 |  |
| hsa-miR-4508 | 14.8828 | 0 | 17.1833 | 0.658385093 |  |
| hsa-miR-4511 | 1.013 | 0 | 13.3063 | 1 |  |
| hsa-miR-4516 | 1.5584 | 0 | 13.9278 | 1 |  |
| hsa-miR-4532 | 5.2986 | 0 | 15.6933 | 1 |  |
| hsa-miR-4533 | 0.8571 | 0 | 13.0653 | 1 |  |
| hsa-miR-378i | 1.7142 | 0 | 14.0653 | 1 |  |
| hsa-miR-1587 | 1.013 | 0 | 13.3063 | 1 |  |
| hsa-miR-3960 | 4.4414 | 0 | 15.4387 | 1 |  |
| hsa-miR-4659a-5p | 0.935 | 0 | 13.1908 | 1 |  |
| hsa-miR-4659a-3p | 1.4026 | 0 | 13.7758 | 1 |  |
| hsa-miR-4659b-5p | 0.7792 | 0 | 12.9278 | 1 |  |
| hsa-miR-4665-5p | 3.9739 | 0 | 15.2783 | 1 |  |
| hsa-miR-4669 | 1.3246 | 0 | 13.6933 | 1 |  |
| hsa-miR-4677-3p | 2.1038 | 0 | 14.3607 | 1 |  |
| hsa-miR-4685-3p | 1.4026 | 0 | 13.7758 | 1 |  |
| hsa-miR-451b | 3.3506 | 0 | 15.0321 | 1 |  |
| hsa-miR-4732-5p | 4.7531 | 0 | 15.5366 | 1 |  |
| hsa-miR-4732-3p | 6.2336 | 0 | 15.9278 | 1 |  |
| hsa-miR-3064-5p | 1.3246 | 0 | 13.6933 | 1 |  |
| hsa-miR-4738-3p | 1.1688 | 0 | 13.5127 | 1 |  |
| hsa-miR-4742-3p | 0.935 | 0 | 13.1908 | 1 |  |
| hsa-miR-4746-5p | 1.948 | 0 | 14.2497 | 1 |  |
| hsa-miR-4750-5p | 0.935 | 0 | 13.1908 | 1 |  |
| hsa-miR-4755-3p | 0.7792 | 0 | 12.9278 | 1 |  |
| hsa-miR-4785 | 1.1688 | 0 | 13.5127 | 1 |  |
| hsa-miR-5001-3p | 1.3246 | 0 | 13.6933 | 1 |  |
| hsa-miR-548ao-3p | 0.7792 | 0 | 12.9278 | 1 |  |
| hsa-miR-548ap-5p | 10.2855 | 0 | 16.6502 | 0.523809524 |  |
| hsa-miR-5010-5p | 0.935 | 0 | 13.1908 | 1 |  |
| hsa-miR-5010-3p | 2.6493 | 0 | 14.6933 | 1 |  |
| hsa-miR-5187-5p | 0.8571 | 0 | 13.0653 | 1 |  |
| hsa-miR-5189-5p | 1.7922 | 0 | 14.1294 | 1 |  |
| hsa-miR-5193 | 0.8571 | 0 | 13.0653 | 1 |  |
| hsa-miR-548aq-3p | 4.3635 | 0 | 15.4132 | 1 |  |
| hsa-miR-664b-5p | 0.8571 | 0 | 13.0653 | 1 |  |
| hsa-miR-548av-5p | 9.74 | 0 | 16.5716 | 0.523809524 |  |
| hsa-miR-548av-3p | 2.7272 | 0 | 14.7351 | 1 |  |
| hsa-miR-5695 | 0.8571 | 0 | 13.0653 | 1 |  |
| hsa-miR-5787 | 0.935 | 0 | 13.1908 | 1 |  |
| hsa-miR-6130 | 1.1688 | 0 | 13.5127 | 1 |  |
| hsa-miR-6131 | 2.8051 | 0 | 14.7758 | 1 |  |
| hsa-miR-6134 | 1.948 | 0 | 14.2497 | 1 |  |
| hsa-miR-6501-5p | 3.1168 | 0 | 14.9278 | 1 |  |
| hsa-miR-6503-3p | 3.3506 | 0 | 15.0321 | 1 |  |
| hsa-miR-6511a-3p | 1.4805 | 0 | 13.8538 | 1 |  |
| hsa-miR-6513-5p | 1.6363 | 0 | 13.9982 | 1 |  |
| hsa-miR-6513-3p | 1.948 | 0 | 14.2497 | 1 |  |
| hsa-miR-6515-5p | 3.1947 | 0 | 14.9634 | 1 |  |
| hsa-miR-6724-5p | 1.2467 | 0 | 13.6058 | 1 |  |
| hsa-miR-6734-5p | 1.013 | 0 | 13.3063 | 1 |  |
| hsa-miR-6741-5p | 2.8051 | 0 | 14.7758 | 1 |  |
| hsa-miR-6741-3p | 1.5584 | 0 | 13.9278 | 1 |  |
| hsa-miR-6747-3p | 1.6363 | 0 | 13.9982 | 1 |  |
| hsa-miR-6772-3p | 1.7142 | 0 | 14.0653 | 1 |  |
| hsa-miR-6806-3p | 0.7792 | 0 | 12.9278 | 1 |  |
| hsa-miR-6807-5p | 0.8571 | 0 | 13.0653 | 1 |  |
| hsa-miR-6819-3p | 1.013 | 0 | 13.3063 | 1 |  |
| hsa-miR-6842-5p | 1.2467 | 0 | 13.6058 | 1 |  |
| hsa-miR-6842-3p | 24.389 | 0 | 17.8959 | 0.282608696 |  |
| hsa-miR-6843-3p | 1.013 | 0 | 13.3063 | 1 |  |
| hsa-miR-6850-5p | 0.7792 | 0 | 12.9278 | 1 |  |
| hsa-miR-6852-5p | 23.6877 | 0 | 17.8538 | 0.282608696 |  |
| hsa-miR-6852-3p | 2.8051 | 0 | 14.7758 | 1 |  |
| hsa-miR-6859-5p | 1.4026 | 0 | 13.7758 | 1 |  |
| hsa-miR-6868-3p | 0.935 | 0 | 13.1908 | 1 |  |
| hsa-miR-6877-5p | 2.8051 | 0 | 14.7758 | 1 |  |
| hsa-miR-6884-5p | 0.7792 | 0 | 12.9278 | 1 |  |
| hsa-miR-7706 | 8.805 | 0 | 16.426 | 0.523809524 |  |
| hsa-miR-4433b-5p | 1.1688 | 0 | 13.5127 | 1 |  |
| hsa-miR-4433b-3p | 16.9087 | 0 | 17.3674 | 0.658385093 |  |
| hsa-miR-1273h-5p | 10.4413 | 0 | 16.6719 | 0.523809524 |  |
| hsa-miR-1273h-3p | 17.9996 | 0 | 17.4576 | 0.430641822 |  |
| hsa-miR-6516-5p | 1.2467 | 0 | 13.6058 | 1 |  |
| hsa-miR-7849-3p | 1.0909 | 0 | 13.4132 | 1 |  |
| hsa-miR-7851-3p | 0.8571 | 0 | 13.0653 | 1 |  |
| hsa-miR-7854-3p | 3.8181 | 0 | 15.2206 | 1 |  |
| hsa-miR-7975 | 0.935 | 0 | 13.1908 | 1 |  |
| hsa-miR-7976 | 13.3243 | 0 | 17.0237 | 0.658385093 |  |
| hsa-miR-7977 | 3.1168 | 0 | 14.9278 | 1 |  |
