# Supplementary figures and images for: Exosomal MiR-1290 Promotes Angiogenesis of Hepatocellular Carcinoma via Targeting SMEK1
Source: J Oncol. 2021 Jan 29;2021:6617700. doi: 10.1155/2021/6617700 (PMC7864765; doi:10.1155/2021/6617700)

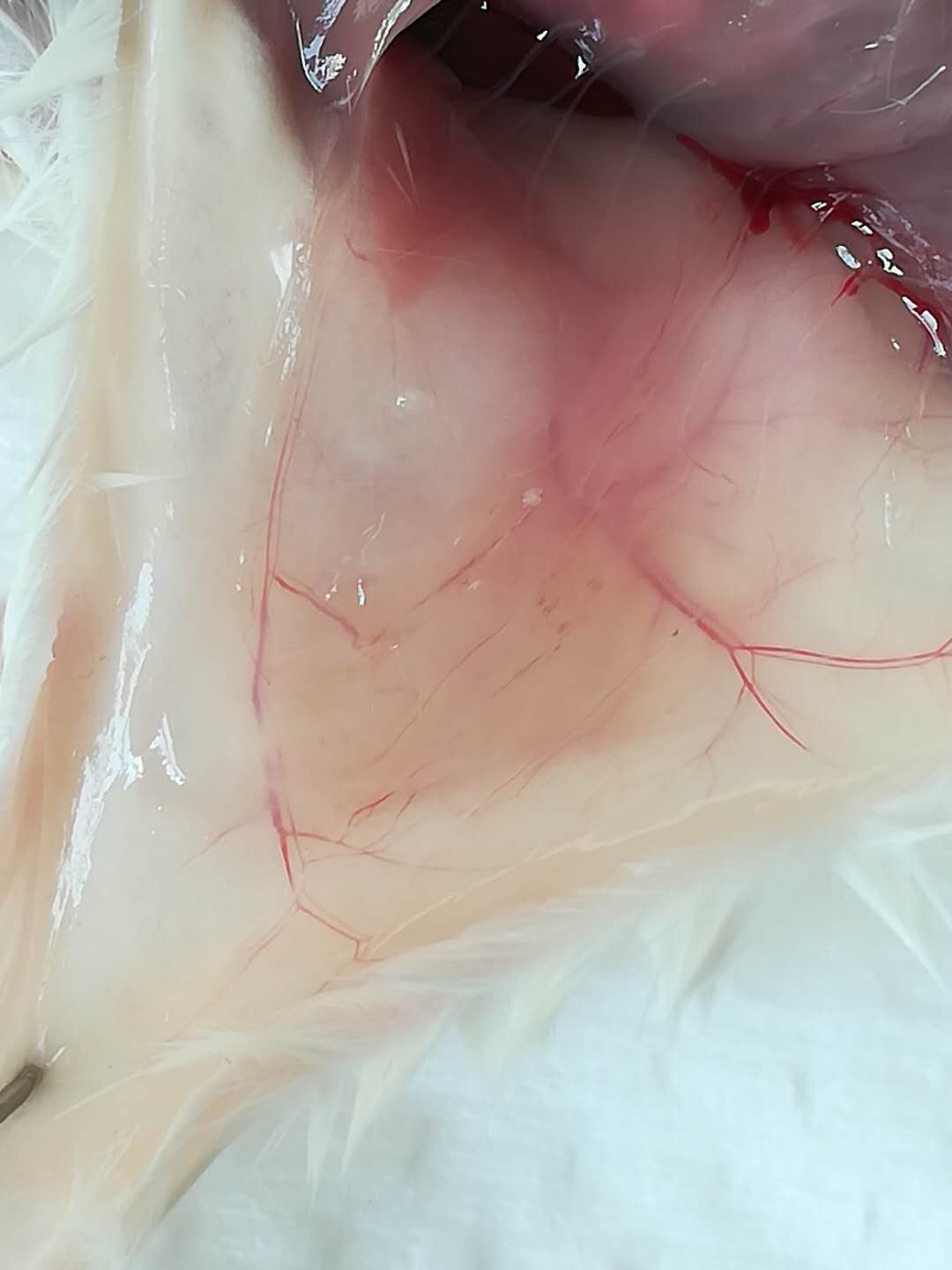

Supplement: Supplementary Materials — Figure S1. miR-1290 targets SMEK1 inSMMC-7721 xenografts Table S1. A list of primers used in the reactions for qRT-PCR. Table S2. A list of primers used in the reactions for clone PCR. Table S3. MiRNA sequencing results. [file 6617700.f1.zip › 6617700.f1/miR-1290-agomir treated Matrigel plug.docx]

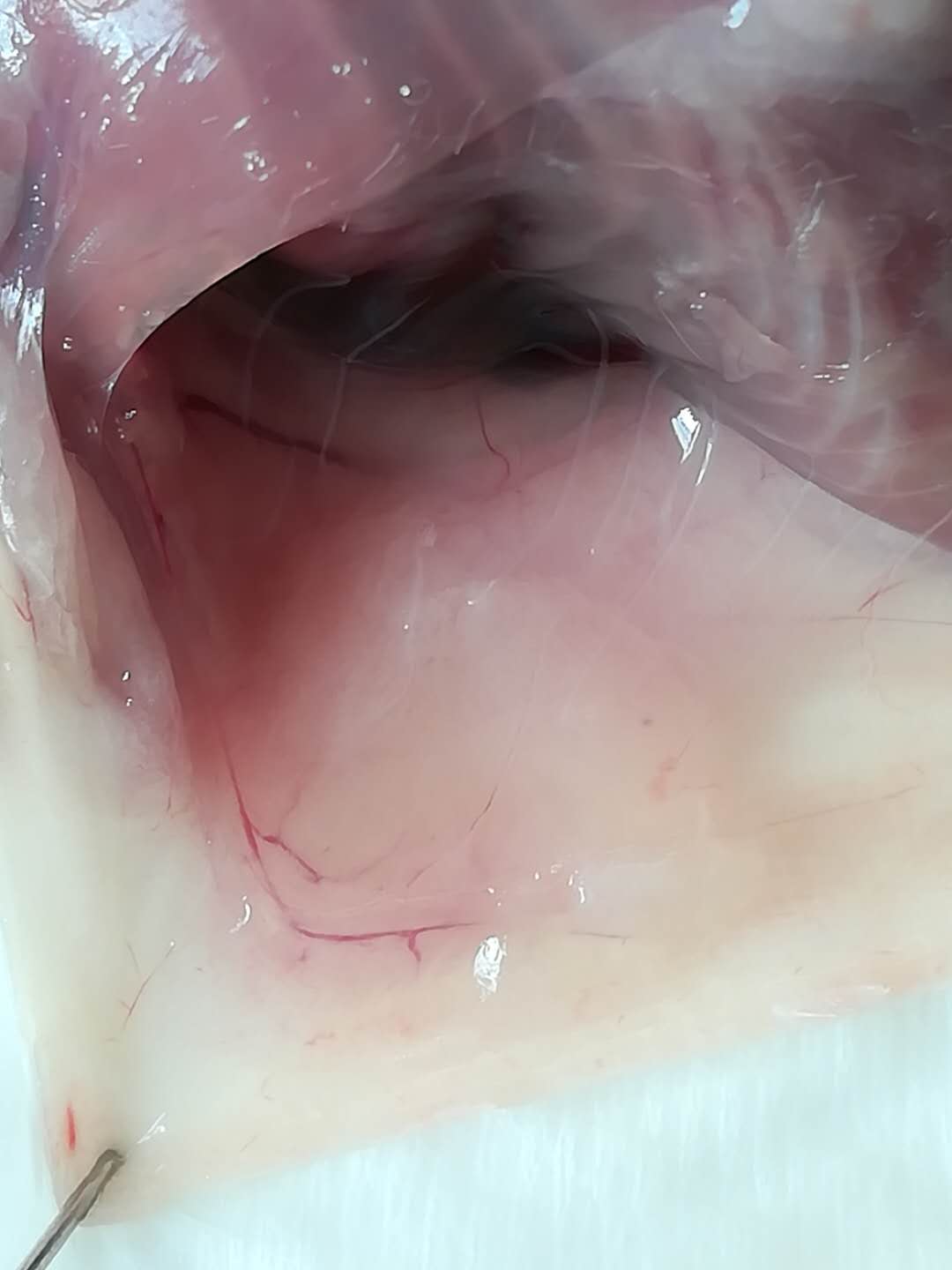

Supplement: Supplementary Materials — Figure S1. miR-1290 targets SMEK1 inSMMC-7721 xenografts Table S1. A list of primers used in the reactions for qRT-PCR. Table S2. A list of primers used in the reactions for clone PCR. Table S3. MiRNA sequencing results. [file 6617700.f1.zip › 6617700.f1/NC-agomir treated Matrigel plug.docx]
